# Effect of Different Frequencies of Transcutaneous Electrical Acupoint Stimulation (TEAS) on EEG Source Localization in Healthy Volunteers: A Semi-Randomized, Placebo-Controlled, Crossover Study

**DOI:** 10.3390/brainsci15030270

**Published:** 2025-03-03

**Authors:** Rael Lopes Alves, Maxciel Zortea, David Mayor, Tim Watson, Tony Steffert

**Affiliations:** 1 Independent Researcher, Porto Alegre 90035-006, Brazil; 2 Independent Researcher, Porto Alegre 90470-230, Brazil; max.zortea@gmail.com; 3 School of Health and Social Work, University of Hertfordshire, Hatfield AL10 9AB, UK; davidmayor@welwynacupuncture.co.uk (D.M.); proftimwatson@gmail.com (T.W.); 4 MindSpire, Napier House, 14-16 Mount Ephraim Rd., Tunbridge Wells TN1 1EE, UK; 5 School of Life, Health and Chemical Sciences, Walton Hall, The Open University, Milton Keynes MK7 6AA, UK

**Keywords:** transcutaneous electroacupuncture stimulation, sham stimulation, EEG, source localization, sLORETA

## Abstract

**Background/Objectives:** Transcutaneous electrical acupoint stimulation (TEAS), also known as transcutaneous electroacupuncture stimulation, delivers electrical pulses to the skin over acupuncture points (“acupoints”) via surface electrodes. Electroencephalography (EEG) is an important tool for assessing the changes in the central nervous system (CNS) that may result from applying different TEAS frequencies peripherally—i.e., acting via the peripheral nervous system (PNS)—and determining how these influence cerebral activity and neural plasticity. **Methods**: A total of 48 healthy volunteers were allocated in a semi-randomized crossover study to receive four different TEAS frequencies: 2.5 pulses per second (pps); 10 pps; 80 pps; and sham (160 pps at a low, clinically ineffective amplitude). TEAS was applied for 20 min to each hand at the acupuncture point Hegu (LI4). The EEG was recorded during an initial 5 min baseline recording, then during TEAS application, and after stimulation for a further 15 min, separated into three periods of 5 min (initial, intermediate, and final) in order to assess post-stimulation changes. Source localization analysis was conducted for the traditional five EEG frequency bands: delta (0.1–3.9 Hz), theta (4–7.9 Hz), alpha (8–13 Hz), beta (14–30 Hz), and gamma (30.1–45 Hz). **Results**: Within-group source localization analyses of EEG data showed that during the initial 5 min post-stimulation, theta oscillations in the 2.5 pps TEAS group increased over the parahippocampal gyrus (t = 4.42, *p* < 0.01). The 10 pps TEAS group exhibited decreased alpha rhythms over the inferior parietal gyrus (t = −4.20, *p* < 0.05), whereas the sham (160 pps) TEAS group showed decreased delta rhythms over the postcentral gyrus (t = −3.97, *p* < 0.05). During the intermediate 5 min post-stimulation, the increased theta activity over the left parahippocampal gyrus (BA27) remained in the 2.5 pps TEAS group (t = 3.97, *p* < 0.05). However, diminished alpha rhythms were observed in the 10 pps TEAS group over the postcentral gyrus (t = −4.20, *p* < 0.01), as well as in the delta rhythms in the sham (160 pps) TEAS group in the same area (t = −4.35, *p* < 0.01). In the final 5 min post-stimulation, reduced alpha rhythms were exhibited over the insula in the 10 pps TEAS group (t = −4.07, *p* < 0.05). Interaction effects of condition by group demonstrate decreased alpha rhythms in the 10 pps TEAS group over the supramarginal gyrus during the initial 5 min post-stimulation (t = −4.31, *p* < 0.05), and decreased delta rhythms over the insula in the sham TEAS group during the final 5 min post-stimulation (t = −4.42, *p* < 0.01). **Conclusions**: This study revealed that low TEAS frequencies of 2.5 pps and 10 pps modulate theta and alpha oscillations over the brain areas related to emotional and attentional processes driven by external stimuli, as well as neural synchronization of delta rhythms in the sham group in brain areas related to stimulus expectation at baseline. It is hoped that these findings will stimulate further research in order to evaluate such TEAS modulation effects in clinical patients.

## 1. Introduction

Electrical stimulation (ES) is a safe and effective technique commonly used to aid recovery of peripheral and central neural circuits after injury [1,2,3]. As observed in animal and clinical studies, plasticity mechanisms promote nerve growth effects, sprouting, and elongation according to the central or peripheral stimulation site [4,5,6,7,8]. Therefore, ES exhibits a regenerative potential for nervous system treatment, and is potentially an attractive therapeutic approach in clinical settings [9].

Several studies have demonstrated that peripheral electrical stimulation (PES) induces cortical plasticity in healthy individuals. In one systematic review of stimulation parameters [10], the intensity of PES was found to be the parameter that exerts the strongest effect on the modulation of motor cortex excitability [11,12]. Furthermore, prolonged ES induced more sustained changes in cortical excitability, supporting the time effect of PES. Few studies were found in which the effects of different PES frequencies were compared, so they were less clear-cut [10].

On the basis of these findings, relationships between the intensity of neuromuscular electrical stimulation (NMES) and brain activity were verified through neuroimaging techniques. NMES can potentially change brain connectivity patterns not only in the somatosensory cortex circuits but also within the motor network [13].

Different techniques have been used to deliver ES to the peripheral nervous system, such as transcutaneous electrical nerve stimulation (TENS), with trains of electrical stimulation delivered via surface electrodes to the skin for pain control, and electroacupuncture (EA), in which electrical pulses are delivered to needles inserted into the acupuncture points [14].

Transcutaneous electrical acupoint stimulation (TEAS) combines TENS and EA techniques in delivering electrical pulses to the skin over acupuncture points via surface electrodes [15]. As with TENS and EA, TEAS uses “low” (<10 Hz) and “high” frequencies (>50 Hz) to promote analgesic and other effects [14]. The same endogenous mechanisms are usually invoked in explaining the pain relief that can result from all these techniques. According to the gate control theory, TENS promotes the segmental inhibition of nociceptive pathways in the dorsal horn of the spinal cord and drives descending inhibitory pathways [16]. In addition, release of endogenous opioids has been suggested to explain the effect of different TENS frequencies that activate specific opioid receptors located in the spinal cord, as well as in the descending inhibition system, including the nucleus raphe magnus in the rostral ventral medulla (RVM) and the periaqueductal gray (PAG) [14]. A brief literature review of publications on the effects of different frequencies of EA, TENS, and TEAS can be found in the Supplementary Materials.

These analgesic frequency-dependent effects were corroborated for a chronic constriction injury (CCI) model of neuropathic pain in rats in which low-frequency TEAS (2 Hz) attenuated mechanical allodynia and thermal hyperalgesia in injured animals [17]. Moreover, this group exhibited significantly increased expression of the mu opioid receptor in the dorsal root ganglia (DRG) [17]. According to functional magnetic resonance imaging (fMRI) studies, the sedative effect of TEAS, measured by a reduced level of consciousness or decreasing pain threshold, was associated with activation of several networks in the brain, such as the default mode network (DMN), sensorimotor network (SMN), and modulation of the function of deep brain areas, including the thalamus [18,19,20].

TEAS has increasing relevance in the clinical setting and has been introduced as an additional treatment technique for various conditions where pain is present. A recent meta-analysis evaluated postoperative pain as the primary outcome using a visual analog scale (VAS) within 24 h after surgery [21]. The main type of surgery investigated was laparoscopy; however, it also included cancer, hemorrhoid resection, and others. TEAS decreased postoperative pain, analgesic consumption after surgery, and the incidence of dizziness, nausea, and vomiting [21]. Considering only laparoscopy surgeries to relieve postoperative pain and reduce analgesic consumption, TEAS shortened hospitalization times and improved patients’ quality of life [22]. Similarly, according to a systematic review and meta-analysis, the main effects of TEAS after total knee arthroplasty (TKA) were postoperative pain alleviation, functional improvement, and a lower incidence of analgesia-related adverse events [23].

In addition to improving pain, TEAS has been demonstrated to be effective in preventing the incidence of postoperative delirium (POD), which is characterized by severe cognitive impairment and alterations in consciousness [24,25,26,27]. Beyond pain treatment, TEAS has shown significant improvement in reproductive medicine related to clinical pregnancy, embryo implantation, and live birth rates [28]. In addition, TEAS applied over cranial and auricular acupuncture points decreased depression symptoms in patients diagnosed with mild-to-moderate major depressive disorder (MDD), showing similar efficacy to the antidepressant medication escitalopram [29].

Related to the above, TEAS has been widely applied in clinical settings, often because of its sedative effects [20]. In this sense, the Hegu acupuncture point, also known as “Large intestine 4” (LI4), is one of the most commonly used acupuncture points for pain and “acupuncture analgesia” [30], and stimulation of the point is thus likely to induce activity changes in the central and autonomic nervous system [31]. The Hegu acupuncture point is positioned on the dorsum of the hand and at the midpoint of the radial side of the second metacarpal bone [32,33]. It is used, for example, to treat chronic back pain, headache, dizziness, toothache, swollen throat pain, facial palsy, and hemiplegia [34,35,36]. A recent meta-analysis evaluating several functional neuroimaging studies in healthy volunteers showed that acupuncture at the Hegu points promotes functional changes between several brain networks, such as the SMN and limbic system [37]. Modulation of these brain regions has also been observed in EA and TEAS neuroimaging studies [36,38,39].

Electroencephalography (EEG) is a noninvasive method of measuring the brain’s electric fields and is thus used as a technique that provides valuable information about the rhythms of the brain, as well as being useful for determining novel therapeutic strategies [40,41]. Resting-state EEG is commonly used to measure abnormalities in the frequency and topographical features of brain oscillations recorded from the scalp during a short period under eyes-open (EO) and eyes-closed (EC) conditions [42].

“Source localization” is one method used to extract information on topographic features from the EEG based on scalp surface recordings in order to estimate current sources within the brain as a function of time [43]. To estimate the current source, source localization analyses need to solve the “inverse problem”, computing cortical sources mathematically in order to determine the relationship between electric potentials from discrete sites on the scalp with inner signals from the brain, according to the geometry and conductivity of regions within the head. This method thus allows an estimation of the location and magnitude of current sources internal to the brain [42,44,45].

As an example, a source localization method was employed in one TEAS study to evaluate whether low (2 Hz)- or high (100 Hz)-frequency stimulation applied at the Hegu acupuncture point modulates certain EEG bands and to determine the underlying current sources. A significant decrease in absolute power was observed in the theta frequency band during high-frequency TEAS, and the topographic source of theta activity was the anterior cingulate cortex (ACC) [46].

Similarly, the effects of “dense-disperse” TEAS (2 Hz and 100 Hz alternating once every 3 s) on the electric activity of the brain in surgery patients anesthetized with propofol showed increased alpha and beta band power during light propofol sedation and reduced delta and beta band power during deep propofol sedation. In addition, synchronization (coherence) between the EEG channels increased at low and decreased at high TEAS frequencies [47].

A previous TEAS study, using advanced machine learning techniques on the same data as analyzed here, demonstrated that the greatest effects on the brain in EEG from baseline occurred in the TEAS group at 80 pps or in the sham group (160 pps), compared with other TEAS frequencies [48].

In a further machine learning study of the same data, the responsiveness of the different EEG frequency bands to TEAS demonstrated that the greatest sensitivity to TEAS occurred in the gamma band, suggesting significant effects on cognitive function. Saliency mapping revealed that the frontal and temporal electrodes provide a more accurate classification, indicating that this subset of electrodes can be used for efficient EEG setups [49]. These results emphasize how EEG might be an important tool to measure modulation in the central nervous system (CNS) promoted by different TEAS frequencies (low or high) applied over the peripheral nervous system (PNS) [50], and how such stimulation promotes the synchronization or desynchronization of different neural clusters [51,52].

Based on the findings described above, we aimed in the present study to determine (a) if comparing post-stimulation with baseline, current sources of traditional EEG frequency bands (theta, delta, alpha, beta, and gamma) were modulated by different frequencies of TEAS (2.5 pps, 10 pps, 80 pps, and sham) applied at a traditional acupuncture point (Hegu) in healthy subjects; and (b) which brain areas were modulated by different TEAS frequencies when compared with the sham group regarding post-treatment to baseline variation, again in the traditional EEG frequency bands.

## 2. Materials and Methods

### 2.1. Study Design and Setting

A crossover study was conducted in which ethics approval was obtained from the Health and Human Sciences Ethics Committee with Delegated Authority of the University of Hertfordshire (UH) (Protocol number HSK/SF/UH/00124), according to international ethical standards based on the Declaration of Helsinki. All participants provided signed written informed consent agreeing that their data would remain anonymous and would be retained for further analysis by the research team, to be shared later with the Human Brain Indices (HBImed) reference database.

### 2.2. Participants, Recruitment, and Inclusion and Exclusion Criteria

**Figure 1:**
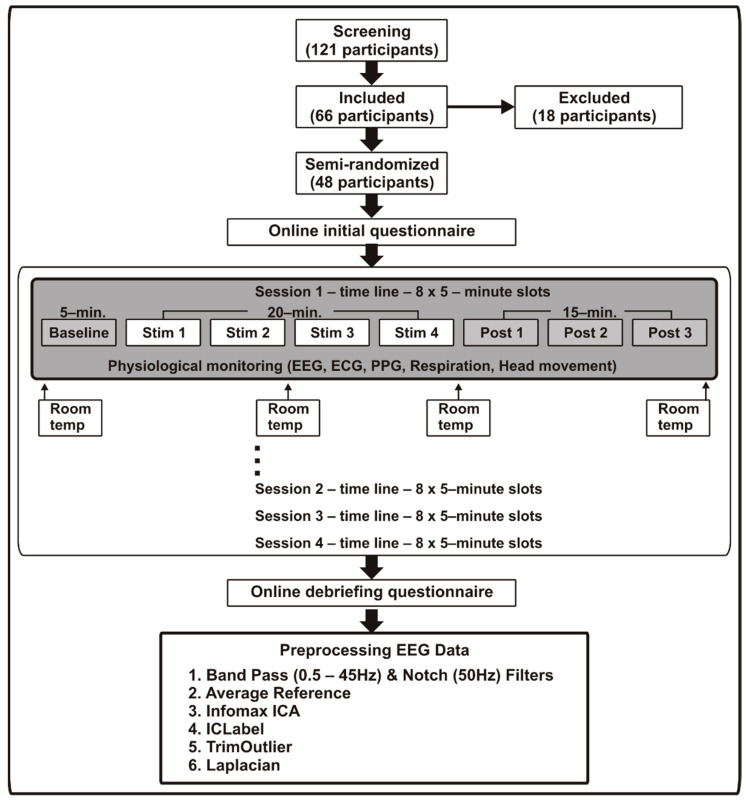
Flowchart of the study assessments. EEG: electroencephalography; ECG: electrocardiogram; PPG: photoplethysmography.

One hundred twenty-one participants were screened from healthy staff and students at the University of Hertfordshire (UH), local complementary health practitioners, and other contacts who volunteered to participate in this study. Sixty-six healthy volunteers participated in the study and completed preliminary online questionnaires. After receiving an explanation of the study protocol, they were required to attend the University Physiotherapy Laboratory for four TEAS sessions, spaced a week or more apart in order to avoid any carryover effect as far as possible [53]. Four participants who dropped out after only one session and another who completed only three sessions were excluded. Another 14 participants were excluded, either because of inadvertent differences in sampling rate (for four participants, in four sessions) or missing or poor-quality data, or because recordings were cut short for one reason or another (e.g., discomfort from wearing the cap or having to take a trip to the bathroom). Thus, 48 subjects were included in the analysis. Figure 1 shows the sequence of the screening, data collection, and preprocessing pipeline.

### 2.3. Intervention

A charge-balanced Equinox E-T388 stimulator was used in all four sessions, and in each session, the device was set at one of four different frequencies: 2.5 alternating monophasic rectangular pulses per second (pps), 10 pps, 80 pps, or 160 pps (the frequency or number of cycles of stimulation per second, in Hertz, was at half these values). The output amplitude was set to provide a “strong but comfortable” sensation for the participant at the three lower frequencies. In contrast, 160 pps was applied as a “sham” treatment, with the device switched on (and a flashing light visible), but the output amplitude remained at zero throughout; although some participants were unable to feel the stimulation at 160 pps, others did indeed feel it as there was some residual current flow despite the output control being set to zero.

**Figure 2:**
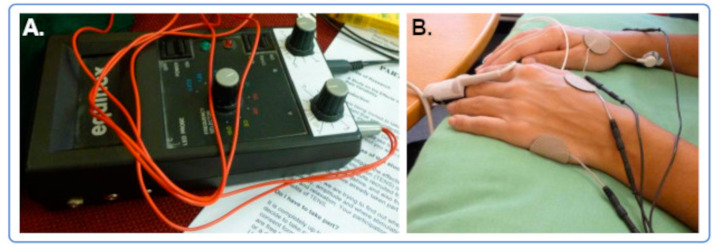
TEAS details. (**A**) The Equinox stimulator. (**B**) Positions of the PPG sensor, and of the ECG and TEAS electrodes.

During 20 min (Stim1–Stim4, or “time slots 2–5”), TEAS was applied to each hand over the acupuncture point Large Intestine 4 (LI4/Hegu), located on the dorsum of the hand between the 1st and 2nd metacarpal bones [30], and the ulnar border of the same hand. This configuration sought to ensure that the current would only pass between the electrodes on each hand and did not flow through the arms and torso, so that, in principle, it should not affect the heart—or brain—directly. Figure 2 shows the details of the TEAS application.

### 2.4. Assessment of the Primary Outcome

All sessions were conducted in the University Physiotherapy Laboratory. This was not completely soundproofed or temperature-controlled, but efforts were made to keep extraneous sounds to a minimum and maintain the temperature in the laboratory within a comfortable range over the several seasons during which data were collected (October 2015–December 2016). Participants remained seated upright in a comfortable chair, with both forearms supported. The session preparation, which took around 15 min, involved the placement of an EEG cap with an attached head movement sensor, electrocardiogram (ECG) electrodes to the forearms, and photoplethysmograph (PPG) and temperature finger sensors on the middle finger of the left hand (see Figure 2). Each session usually lasted around 60–90 min. EEG and other data were collected during all procedures (8 × 5-min “slots”) per session: the initial 5 min of baseline recording (“time slot 1”), the following 20 min of TEAS application (Stim1–Stim4, or “time slots 2–5”), and a further 15 min after stimulation (Post1–Post3, or “slots 6–8”) to assess post-stimulation changes. The data collection followed standard EEG procedures using Electro Cap International (ECI) caps (size selected individually for maximum comfort, according to participants’ head dimensions), a Mitsar EEG-202 amplifier, and WinEEG software (v2.91.54) (Mitsar Ltd., St. Petersburg, Russia) [48]. EEG was recorded using 19 electrodes located on Fp1, Fp2, F7, F3, Fz, F4, F8, T3, C3, Cz, C4, T4, T5, P3, Pz, P4, T6, O1, and O2, with linked ears as reference and ground anterior to Fz [54]. The sampling rate was 500 Hz [48].

### 2.5. Preprocessing and Functional Connectivity Analysis

The collected EEG data were recorded in WinEEG format (“.eeg”). All EEG data preprocessing steps were performed using the open-source toolbox EEGLAB version 14.1.1b [55]. Each session generated one file that was saved in “.edf” format and sliced into eight separate “.mat” files, each related to one slot with a duration of 5 min. Thus, we applied two filtering methods. First, the data were filtered between 0.5 and 45 Hz using second-order Butterworth filters (in MATLAB version 9.8 and released number R2020a, using the “butter” and “filtfilt” functions), so that they were processed in both forward and reverse directions in order to achieve zero phase delay. However, due to the slow roll-off rate of the second-order Butterworth filter, in addition, we next applied, for higher-frequency signals, a second-order 50 Hz Butterworth notch filter between 49 and 51 Hz (mains power in the UK is supplied at 50 Hz). The reference to average was also added. To reject non-neural artifacts, we conducted an Independent Component Analysis (ICA), applying the extended Infomax ICA algorithm as implemented in the pop_runica function; however, the “PCA” option was disabled [56]. To correct for rank deficiency, we used the approach of adding an additional channel filled with zeros before re-referencing and removing the additional channel afterwards [57]. The components were labeled using ICLabel [58], and artifact components were selected and removed according to the parameter settings. The number of retained components was defined as the maximum number of available channels. Muscle movements, eye blinks, heart beats, and line noise were considered artifacts exceeding the 50% threshold. Channel noise was considered an artifact if it exceeded the 30% threshold. In addition to the artifact rejection thresholds, the probability of the component being related to brain activity had to be less than 10% for eye blinks and muscle movements, and less than 5% for all remaining categories. Meeting both conditions was required for good data rejection performance. After removing the artifacts, the maps were saved, and a new set of data files was created. The Trimoutlier EEGLab plugin was then used to clean the raw data and withdraw epochs that exceeded an amplitude threshold of ±3 SD (standard deviation) above the mean amplitude across all channels [59]. Finally, we reconstruct the data using the CSD Toolbox (ExtractMontage. m function) [60], applying the Laplacian form of the local average reference. Considering that 48 participants remained, with data collected in 4 sessions for each, 192 files per participant were generated (saved in “.mat” format), multiplied by 8 slots, totaling 1536 files.

After the EEG preprocessing steps and artifact removal, we sought to determine the current sources. Source localization analysis refers to the process used to estimate the current sources in the brain from measured EEG data, applying computational methods to solve the inverse problem [43]. The standardized low-resolution brain electromagnetic tomography (sLORETA) method was used to determine the source localization distribution based on images of standardized current density [61,62].

The inverse solutions can be obtained using some form of regularization technique as the cost function [44]. sLORETA employs the standardized minimum norm estimation solution as a regularization technique. It requires variance estimates of current density, thus taking into account the actual source variance as well as the noise variability [44,61]. The LORETA-Key software version 20200709 is freely available at https://www.uzh.ch/keyinst/NewLORETA/Software/Software.htm, accessed on 9 July 2020.

The sLORETA software uses a 3-layer spherical head model based on an anatomical MRI template from the Montreal Neurological Institute (MNI152) to promote slicing and classification of the cortical volume in 6239 voxels of dimension 5 mm^3^ (limited to the cortical gray matter and hippocampus areas) [63,64,65]. Neuronal clusters were classified according to the 43 human functional cortical areas, denominated Brodmann’s Areas (BAs), which correlate cytoarchitectural information with neuronal circuitry, molecular organization, genetics, and individual functional aspects [66].

The source localization maps were generated using the free toolbox BrainNet Viewer version 1.6, which is an easy, flexible, and quick way to visualize theoretical networks. This software was created in the MATLAB programming language and is available on the Neuroimaging Informatics Tools Resources Collaboratory website. (NITRC): www.nitrc.org/projects/bnv/ [67], accessed on 4 January 2023.

The following steps were employed to determine source localization across the five frequency bands:

Step 1: Within-group analyses of source localization were performed. Paired groups analyses were performed under the following conditions: baseline (Slot 1) and post-stimulation time (Slots 6, 7, and 8) with regard to the distinct TEAS frequency groups (2.5 pps, 10 pps, 80 pps, and 160 pps). In other words, we assessed 2.5 pps (Slot 6) vs. 2.5 pps (Slot 1), 10 pps (Slot 6) vs. 10 pps (Slot 1), 80 pps (Slot 6) vs. 80 pps (Slot 1), and 160 pps (Slot 6) vs. 160 pps (Slot 1). The same procedure was followed for slots 7 and 8.

Step 2: Source localization analyses of interaction effects of condition by group were executed. We examined for each post-stimulation time the difference between the TEAS frequencies (Slots 6, 7, and 8) and baseline (Slot 1), and compared these differences with the corresponding difference in the sham group (160 pps). The dependent group test was conducted to determine source localization according to the following formula: [2.5 pps (Slot 6) − 2.5 pps (Slot 1)] vs. [160 pps (Slot 6) − 160 pps (Slot 1)], [10 pps (Slot 6) − 10 pps (Slot 1) vs. 160 pps (Slot 6) − 160 pps (Slot 1)], and [80 pps (Slot 6) − 80 pps (Slot 1)] vs. [160 pps (Slot 6) − 160 pps (Slot 1)]. The same procedure was followed for slots 7 and 8.

### 2.6. Statistical Analysis

Descriptive statistics were used to summarize the demographic and clinical features of the sample. Values are presented as mean and standard deviation (SD) or relative frequencies (RFs) and percentages.

Significant source localization differences in the spectral power density between the TEAS frequency groups were determined by comparing the differences between the post-stimulation and baseline EEG recordings. sLORETA statistical contrast maps were calculated for the 5 frequency bands delta (0.1–3.9 Hz), theta (4–7.9 Hz), alpha (8–13 Hz), beta (14–30 Hz), and gamma (30.1–45 Hz). Utilizing a nonparametric permutation test, we computed the permutation distribution to correct for multiple comparisons and calculated the significance level based on the homogeneous distribution generated by the randomized permutation of the data [60,67,68,69]. Estimates of the mean difference normalized by the variance between each voxel and its neighbors formed the t-statistic images. The average variance estimates around the voxel could be obtained by smoothing the raw variance. The brain cortical voxels with significant differences were identified by statistical nonparametric mapping (SnPM) according to a randomization test with 5000 permutations. For all analyses, a two-sided *p* value of 0.05 was considered. The sLORETA software was used to run the analysis [68,69,70].

Any statistical analysis of the sources will suffer from the natural variance between individuals in a given population, which might be due to scalp and skull conductivity varying between subjects. To eliminate variability, a typical procedure consists of scaling (or normalization) any estimated dipole current density in each voxel and frequency band to suppress any sources of variance that are not of physiological interest. Subject-wise normalization scales all frequencies of interest and voxels of the brain volume by a single factor, the mean or the sum of the dipole current density computed. The source solutions are represented by a single normalized factor which eliminates a global source of variability [23]. The normalization (scaling) of the sLORETA images can be represented as s = (b,v), where b denotes the bands (delta; theta; alpha; beta; and gamma); and v denotes the voxel (1 to 6239) [71]. The formula representing the grand average over all frequency bands and voxels (i.e., subject-wise) normalization is presented as follows:

$$\hat{s}\left(b,v\right)=\frac{1}{\left(5\cdot 6239\right)}\sum_{b=1}^{5}\sum_{v=1}^{6239}s\left(b,v\right)$$

## 3. Results

### 3.1. Demographic and Clinical Characteristics of the Sample

Table 1 provides an overview of the demographic and clinical characteristics of the sample.

### 3.2. Brain Activity Within-Group TEAS Frequencies

**Figure 3:**
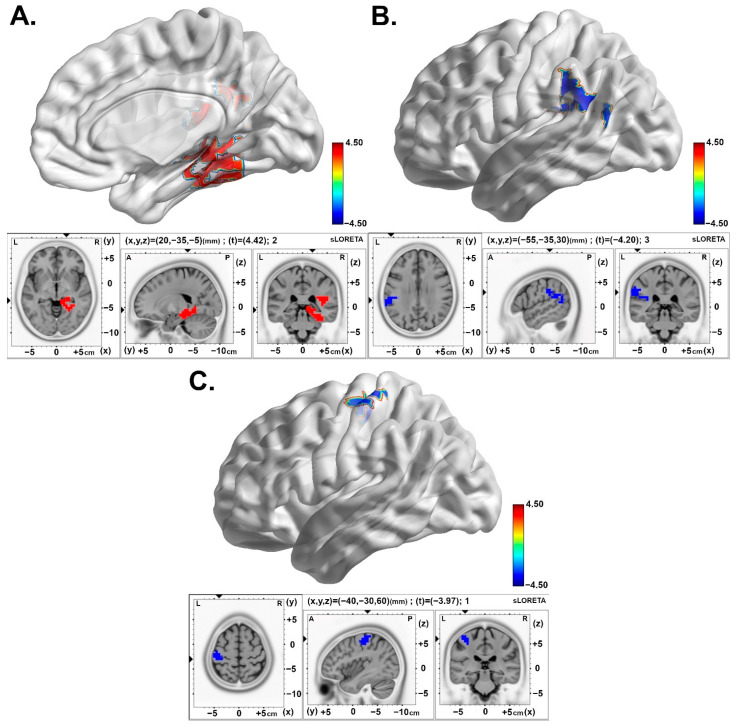
Statistical maps of cortical oscillations in the initial 5 min post-stimulation (Post1). The colored area represents the voxels with the highest significance. (**A**) Increased theta rhythms in the 2.5 pps TEAS group over the parahippocampal gyrus which present a medium to large effect size (Cohen’s d = 0.65). Values are presented as t-values (significance threshold Post > Baseline: t = 3.720, *p* < 0.05). (**B**) Decreased alpha rhythms in the 10 pps TEAS group over the inferior parietal lobe with a medium to large effect size (Cohen’s d = 0.61). Values are presented as t-values (significance threshold Post < Baseline: t = −3.695, *p* < 0.05). (**C**) Decreased delta rhythms in the sham (160 pps) TEAS group over the postcentral gyrus with a medium to large effect size (Cohen’s d = 0.58). Values are presented as t-values (significance threshold Post < Baseline: t = −3.707, *p* < 0.05). Effect sizes are based on Cohen’s d: small = 0.2, medium = 0.5, large = 0.8. L, left; R, right.

In the post-stimulation time (Slot 6) compared with baseline (Slot 1), the 2.5 pps TEAS group showed increased theta band activity (or equivalently, a larger number of synchronously oscillating neurons) in the right parahippocampal gyrus (BA27). The 10 pps TEAS group showed decreased activity in the alpha frequency band of the left inferior parietal lobe (BA40). The sham TEAS group (160 pps) showed decreased delta oscillations activity in the left postcentral gyrus (BA03). The results are presented in Table 2 and Figure 3.

**Figure 4:**
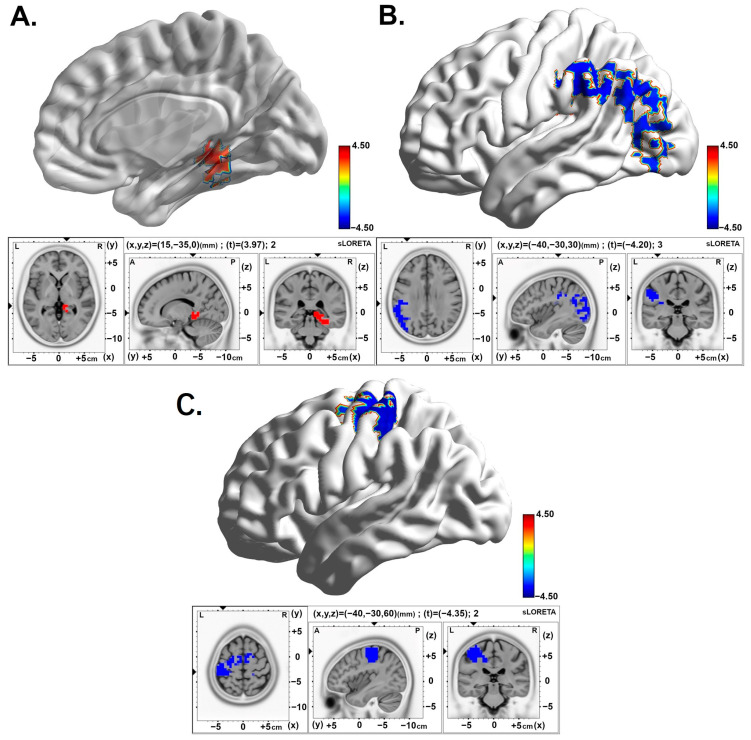
Statistical maps of cortical oscillations during the intermediate 5 min post-stimulation (Post2). The colored area represents the voxels with the highest significance. (**A**) Increased theta rhythms in the 2.5 pps TEAS group over the parahippocampal gyrus with a medium to large effect size (Cohen’s d = 0.58). Values are presented as t-values (significance threshold Post > Baseline: t = 3.688, *p* < 0.05). (**B**) Decreased alpha rhythms in the 10 pps TEAS group over the postcentral gyrus that exhibit a medium to large effect size (Cohen’s d = 0.61). Values are presented as t-values (significance threshold Post < Baseline: t = −3.688, *p* < 0.05). (**C**) Decreased delta rhythms in the sham (160 pps) TEAS group over the postcentral gyrus with a medium to large effect size (Cohen’s d = 0.64). Values are presented as t-values (significance threshold Post < Baseline: t = −3.735, *p* < 0.05). Effect sizes are based on Cohen’s d: small = 0.2, medium = 0.5, large = 0.8. L, left; R, right.

Comparing the post-stimulation time (Slot 7) with baseline (Slot 1), an increase in theta oscillations remains over the right parahippocampal gyrus (BA27) in the 2.5 pps TEAS group. However, the 10 pps TEAS group and the sham TEAS group (160 pps) demonstrated decreased activity in the alpha and delta bands in the left postcentral gyrus (BA02 and BA03), respectively. The results are presented in Figure 4A–C and in Table 2.

**Figure 5:**
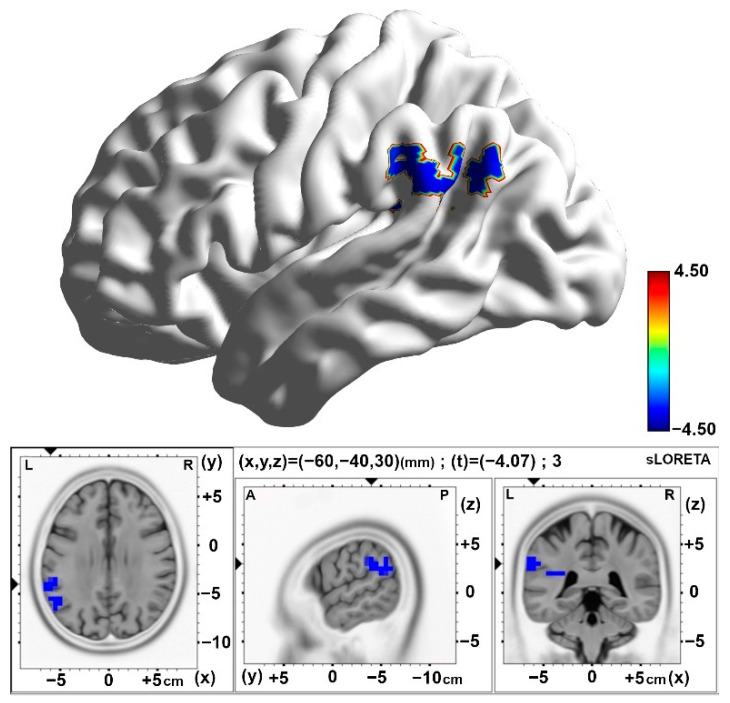
Statistical maps of cortical oscillations in the final 5 min post-stimulation (Post3), showing decreased alpha rhythms in the 10 pps TEAS group over the left inferior parietal lobe with a medium to large effect size (Cohen’s d = 0.60). The colored area represents the voxels with the highest significance. Values are presented as t-values (significance threshold Post < Baseline: t = −3.779, *p* < 0.05). Effect sizes are based on Cohen’s d: small = 0.2, medium = 0.5, large = 0.8. L, left; R, right.

Similarly, comparing post-stimulation time 3 (Slot 8) with baseline (Slot 1), decreased alpha band activity was observed over the left insula (BA13) in the 10 pps TEAS group. See Table 2 and Figure 5.

### 3.3. Interaction Effects of Brain Activity According to Condition by Group

**Figure 6:**
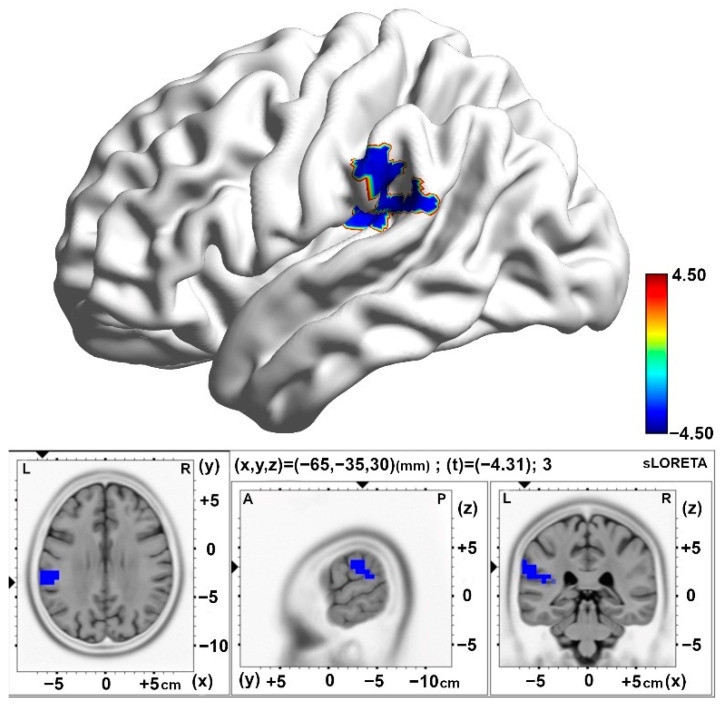
Statistical maps of cortical oscillations in the initial 5 min post-stimulation (Post1), showing decreased alpha rhythms in the 10 pps TEAS group over the inferior parietal lobe, with a medium to large effect size (Cohen’s d = 0.63). The colored area represents the voxels with the highest significance. Values are presented as t-values (significance threshold, Post-Baseline < Post-Baseline): t = −3.744, *p* < 0.05). Effect sizes are based on Cohen’s d: small = 0.2, medium = 0.5, large = 0.8. L, left; R, right.

**Figure 7:**
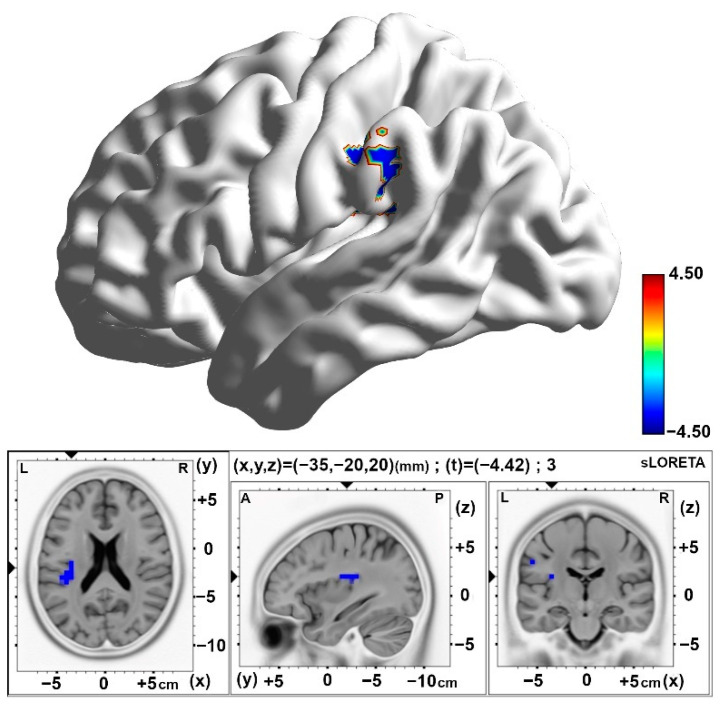
Statistical maps of cortical oscillations in the final 5 min post-stimulation (Post3), showing decreased alpha rhythms in response to 10 pps over the insula with a medium to large effect size (Cohen’s d = 0.65). The colored area represents the voxels with the highest significance. Values are presented as t-values (significance threshold, Post-Baseline < Post-Baseline): t = −3.791, *p* < 0.01). Effect sizes are based on Cohen’s d: small = 0.2, medium = 0.5, large = 0.8. L, left; R, right.

In the differences between the post-stimulation time (Slot 6) and baseline (Slot 1) periods, the 10 pps TEAS group demonstrated significantly decreased activity in the alpha band located in the superior marginal gyrus (BA40) compared with the sham group (Figure 6 and Table 3). Similarly, diminished activity in the alpha band was observed over the insula region (BA13) in the 10 pps TEAS group when differences were analyzed between the post-stimulation time (Slot 8) and baseline (Slot 1). Results are presented in Table 3 and Figure 7.

## 4. Discussion

A finding in this source localization analysis that should be highlighted was that the 10 pps TEAS group modulated alpha oscillations in distinct parietal brain areas in all post-stimulation periods measured in different slots. In the initial and final 5 min post-stimulation periods (Post1 and Post3), decreased alpha frequency oscillations were observed in clusters localized in the inferior parietal gyrus (BA40) extending into the insula and the postcentral gyrus. However, during the intermediate 5 min post-stimulation period (Post2), decreased alpha frequency band was observed in the neural cluster localized in the postcentral gyrus (BA02), extending to the inferior parietal gyrus and insula. Further analyses showed that 2.5 pps TEAS increased theta frequency oscillations with the cluster centered over the parahippocampal gyrus (BA27) during the first and second post-stimulation periods (Post1 and Post2), but this modulated activity did not persist in the final period.

Importantly, we underline the modulation that occurred in the sham group (160 pps) in the initial and second post-stimulation periods (Post1 and Post2), with decreased delta oscillation in a cluster centered over the postcentral gyrus (BA03), extending into other somatosensory areas.

Furthermore, between-group analyses showed decreased alpha oscillations in the 10 pps TEAS group compared with sham (160 pps). The cluster was localized over the superior marginal gyrus (BA40) in the initial 5 min post-stimulation (Post1), extending to the superior temporal gyrus, and the other cluster was located in the insula area (BA13) in the final 5 min post-stimulation period (Post3).

The current study demonstrated that the 10 pps TEAS group was the only group that exhibited sustained modulation during all post-stimulation periods, decreased alpha frequency waves in the inferior parietal gyrus (IPG) (BA40) in the first and last post-stimulation periods, and diminished alpha frequency oscillations over the postcentral gyrus (BA02) during the intermediate post-stimulation period. The modulation occurs over the parietal lobe (PL), which comprises the somatosensory cortex and posterior parietal cortex (PPC). These areas are involved in multiple brain processes, such as transforming sensory inputs into motor responses and selective attention that filters input information for subsequent preferential processing [72].

The PPC is a major associative region in the cortex of the mammalian brain, integrating inputs from several cortical areas and proprioceptive and vestibular signals from subcortical areas. In humans, the PPC comprises the superior parietal gyrus (BA05 and 07), angular gyrus (BA39), and IPG (BA40) [73,74]. Each of these regions participates in multiple functions such as sensory–motor integration, motor planning, spatial attention, spatial navigation, language, decision making, working memory, and number processing [75,76].

The ventral region of the PPC is covered by the IPG, which is organized in two greater gyri, the supramarginal gyrus (SMG; BA40) localized rostrally, and the angular gyrus (AG; BA39) caudally. There is a correspondence between this IPG partition and different cerebral processes and functional networks. Much evidence regarding the anatomy and function of the IPG has been obtained from animal experiments. In monkeys, the rostral areas of IPG were associated with sensory–motor integration, whereas the caudal part was associated with spatial attention. In humans, the rostral IPG is related to motor planning, action-related functions, and the mirror neuron system, while the AG, the caudal part, is related to language and spatial attention [75].

These functions were correlated with the connectivity patterns of the IPG that occur via the superior longitudinal fasciculus [77]. Rostral IPG are strongly connected with the inferior frontal, motor, premotor, and somatosensory areas, whereas caudal IPG is more connected with the lateral and medial prefrontal, posterior cingulate, parahippocampal gyrus, and higher visual and temporal areas [75,78]. In particular, the more rostral areas of IPG are classified as higher somatosensory areas because they exhibit connections with the somatosensory cortex, premotor area, prefrontal cortex, auditory cortex, and insula [74,79]. Some of these areas correspond to EEG source regions modulated by 10 pps TEAS in this study, such as the IPG (BA40), postcentral gyrus (BA02), and insula (BA13). These results indicate that rostral IPG networks may have been modulated by a 10 pps TEAS frequency.

In a recent meta-analysis, traditional acupuncture was found to modulate these areas when applied over acupuncture point LI4 in healthy volunteers. Several regions of the parietal lobe, such as the IPG, somatosensory area, and limbic region, were activated [37]. Likewise, EA over LI4 induced IPG, somatosensory, and limbic system activation in healthy subjects [36]. Such networks were also modulated by TEAS in patients with Bell’s palsy and Alzheimer’s disease [38,80]. Deactivation of several brain areas, including the IPG, was observed after traditional acupuncture application at the LI4 acupuncture point compared with sham stimulation in healthy volunteers in an fMRI study. Furthermore, LI4 acupuncture point stimulation resulted in more deactivated brain areas than other acupuncture points, which may indicate an analgesic effect related to the LI4 acupuncture point [81].

In addition to brain activity modulation measured by fMRI, peripheral nervous system stimulation also changes brain wave patterns. In a spatial-selective attention task, tactile stimulation of the right finger resulted in increased gamma-band power in the contralateral somatosensory cortex, followed by suppression of low-frequency activity (alpha and beta band) in the parietal areas that extended into the bilateral occipital areas [82]. These results demonstrate that spatial-selective attention enhances neuronal responses in somatosensory areas related to processing tactile patterns. Furthermore, the effects of selective attention on stimulus-related activity also recruited visual cortex areas, mainly for attended compared with unattended stimuli [82].

The attentional network is divided into two cortical systems, the ventral and dorsal networks, which are functionally specialized and perform specific attentional control [83]. The ventral network promotes bottom–up attention, which is control-driven by external factors (stimulus-driven), and the dorsal network is oriented toward top–down attention, which is driven by internal (goal-directed) factors. The visual system has served as a reference for attention network studies, but any modality of sensory stimulation might be mediated by these factors [83]. Thus, the direction of attention for the tactile stimulus may be guided by an internal reference (e.g., the location on the skin, organized in the somatosensory homunculus) or oriented by an external cue (e.g., the body posture referenced from the external space) that represents the location at which touch is expected [84].

The brain regions involved in top–down and bottom–up modulation related to the bodily attention component for acupuncture point stimulation demonstrated significant activation of the insula, operculum, supplementary motor area (SMA), primary (SI) and secondary sensory (SII) areas, SMG, PPC, ACC, and fusiform areas, as well as deactivation in the DMN areas, such as the medial prefrontal cortex (MPFC), posterior cingulate cortex (PCC), medial temporal gyrus (MTG), IPG, and parahippocampal regions [85]. 

Based on these observations, a possible neural mechanism to explain the PES modulation of IPG and somatosensory areas by the alpha frequency band activity might be related to attentional control via bottom–up and top-down factors. Alpha frequency plays an important role in setting the state of the somatosensory cortex to improve the processing of tactile stimuli. In the prestimulus period, decreased contralateral and increased ipsilateral alpha activity reflect the lateralized attention control in the somatosensory system and suggest that alpha activity is related to stimulus anticipation [86].

The anticipation of lateralized tactile stimuli improves perception and changes neural activity. In a perceptual identification task, less alpha and beta frequency band activity was observed in the contralateral compared with the ipsilateral sensorimotor cortex [87,88]. Moreover, decreased oscillatory alpha power relative to baseline can be modulated by tactile stimulation and attention. This is endorsed by a TENS study, in which stimulation was applied to two finger areas to evaluate tactile sensations for different vibration frequencies and pressure intensities [89]. Brain oscillatory alpha power decreased over the contralateral somatosensory and prefrontal cortices, particularly in response to vibration at 40 Hz when compared with 20 or 30 Hz. These results indicate that contralateral somatosensory alpha activity might be used as a biomarker to assess tactile perception modulated by top–down pathways [89]. Thus, the somatosensory cortex and alpha frequency may be regarded as the brain structure and oscillation signature, respectively, of the attentional control via the top–down factor of tactile sensory stimuli.

The mechanism implicated in the modulation of brain regions related to the bottom–up attentional process also influences neuronal activity. A powerful stimulus that activates the attention circuitry decreases neural firing variability in multiple brain areas, increases local neuronal synchronization in the visual attention system, and supports the view that bottom–up attention affects neuronal modulation and the transmission of information in the brain [83].

Bottom–up attention processing revealed by neuroimaging studies indicates that the IPG (BA39 and BA40) in the PPC are activated by salient visual stimuli [75]. Moreover, another brain oscillation study revealed that alpha frequency band activity in the PPC is closely related to external–spatial tactile processing according to tactile stimulation tasks in both sighted and congenitally blind participants [90], as well as corroborating the involvement of ipsilateral PPC regions in attention-related tactile processing [91].

In this sense, changes in alpha neural activity over the parietal cortex are related to the modulation of stimulus-driven reorienting of attention. This relationship was corroborated by an experimental study in which Transcranial Magnetic Stimulation (TMS) (10 Hz) applied over the PPC of healthy subjects impaired tactile detection assessed by alpha activity over the contralateral somatosensory cortex and improved tactile stimuli perception in the ipsilateral somatosensory cortex [92]. These results provide evidence for the functional role of alpha oscillation in the PPC as a form of coding tactile representation (tactile attention) of spatial information [92].

Similarly, this relationship was observed in the clinical group in a TENS study with oncological patients who showed decreased alpha power oscillations during the interval before the target stimulus. This outcome reflects the electrical changes over the parietal areas, which are oriented and related to attention to TENS sensory stimuli, and might indicate that decreased alpha oscillations are related to brain analgesic effects [93].

Alpha brain oscillations are hallmarks of relaxation states (i.e., with no directed task), as revealed by EEG/FMRI during resting states. Blood oxygenation level-dependent (BOLD) imaging changes, for example, have been associated with spontaneous alpha rhythms. Moreover, several brain areas, such as the thalamus, ACC, fusiform gyrus, and dorsolateral prefrontal cortex, are correlated with alpha wave activation or deactivation during the resting state [94,95].

In a single-blinded crossover study, 20 healthy volunteers received traditional acupuncture at the LI4 acupuncture point or placebo. The treatment group exhibited increased low-alpha band power in the occipital area, corroborating the relaxation effect related to the enhancement of alpha brain oscillations [31]. A TEAS study that evaluated brain oscillations demonstrated increased power in the low-alpha frequency band (alpha-1) during and after stimulation compared with baseline, independent of TEAS group features (active to sham, or high (100 Hz) to low (2 Hz), TEAS) [46].

The outcomes observed in the present study indicate decreased alpha waves after 10 pps TEAS, occurring at different periods over the parietal areas, specifically IPG (BA40), and the somatosensory cortex (BA02). Alpha rhythm alterations over the parietal cortex indicate bottom–up attentional control driven by cross-modal stimuli [96]. Likewise, the postcentral gyrus (BA02), which performs sensory processing from various parts of the body, also participates in oriented attention and contributes to emotional regulation through attentional strategies [97]. These findings provide evidence that alpha band dynamics are involved in the interconnectivity between the sensory cortex and frontoparietal attention networks [96].

The results of the interaction effects of condition by group showed decreased alpha waves over the insular cortex (BA13) in the 10 pps TEAS group compared with the sham group (160 pps). The anterior insula combined with the IPG, and the temporoparietal junction (TPJ) with the inferior frontal gyrus (IFG), comprise the supramodal network response to visual, sensory, and auditory stimuli. This combination was associated with the control of bottom–up attention to salient visual stimuli [74]. Thus, we hypothesized that the interaction effect results corroborate the modulation hypotheses from the 10 pps TEAS frequency on bottom–up attentional circuitry.

In addition, we found diminished delta frequencies over the postcentral gyrus (BA03) in the sham (160 pps) TEAS group. The relationship between delta waves and sensory information was demonstrated using a combined fMRI/EEG study. Lower EEG frequencies, such as the delta band, displayed a positive association with resting-state networks related to somatosensory cortices but a negative association with resting-state networks related to cognitive functions [98]. These results demonstrate that delta oscillations are associated with motivational/attentional processes that perform a constant screening of internal and external salient stimuli [99].

The motivational relevance of salient stimuli has also been observed in the P300 paradigm, which shows that the enhancement of delta EEG power is positively correlated with increased P300 amplitude in response to salient stimuli [100]. This interpretation suggests that delta oscillations play an important role in the synchronization of brain activity related to several brain functions, such as autonomic, motivational, and cognitive processes [99].

Stimulus processing involves two complementary mechanisms, attention and expectation, related to motivational relevance and previous experience, respectively. Attention and expectation are supramodal processes, whereas spatial attention has a stronger effect in the visual domain and temporal expectation has a more prominent effect in the auditory domain [101]. However, temporal expectation plays an important role in mediating the effects of anticipation on stimuli detection. Anticipation of an external stimulus sets the sensory system’s state, improving neural and behavioral responses. In this sense, delta oscillations play a functional role in anticipatory mechanisms that modulate brain areas responsible for enhancing sensory processing information [102].

A recent meta-analysis showed that manual acupuncture at LI4 promotes activation of the postcentral gyrus, the brain area responsible for sensory processing of information, and discriminates painful stimuli according to their level, location, and duration [37]. The possible explanation for the sham TEAS results suggests that somatosensory networks in this group were modulated through the local synchronization of delta waves related to the anticipation effect for the TEAS application triggered by the stimulus expectation process in the baseline compared with post-stimulation period.

We also verified increased theta oscillation in the parahippocampal gyrus (BA27) in the 2.5 pps TEAS group. In humans, theta waves have been observed in several brain structures, many of which are present in the limbic system. A common theme in published studies is that hippocampal theta rhythm is implicated in episodic memory formation and spatial navigation. Theta power increases during memory encoding after word recall, and according to neuroimaging studies, the hippocampus is the brain area related to episodic memory formation [103].

In an fMRI study, BOLD signal changes were positively correlated with theta band local field potentials (LFPs) measured by depth electrodes implanted in patients for seizure monitoring [104]. Similarly, an EEG/fMRI study showed that theta rhythms arise in the parahippocampal region and associated limbic structures during hippocampal memory encoding [105]. A magnetoencephalography study on memory encoding evaluated goal-directed navigation in healthy subjects and revealed through source analyses greater theta activity in the hippocampus and parahippocampal cortices [106]. Healthy volunteers who received high-frequency (100 Hz) TEAS applied over the LI4 acupuncture point in source localization analyses showed increased theta activity in the midline frontal area after stimulation [46]. This appeared to originate from the cingulate cortex, demonstrating the modulation by high-frequency TEAS of limbic activity [46]. This explanation may be applicable to our study. The synchronized theta oscillation observed over the parahippocampal area in response to 2.5 pps TEAS corresponds to TEAS modulation in the limbic regions related to the emotional component of salient stimuli.

Our findings indicate a possible TEAS modulation of the top–down and bottom–up perception mechanisms, with relevance to the treatment of pain, anxiety, depression, and attention-deficit/hyperactivity disorder (ADHD) in the clinical setting.

Top–down attention processing to pain stimuli induced a decrease in alpha and an increase in gamma-band power localized in the insula, as well as bottom–up modulation of gamma oscillation localized in the sensory–motor and cingulate cortices [107]. These attentional mechanisms related to the pain response provide complementary information about pain perception [108]. Thus, TEAS has a role as a nondrug treatment that promotes analgesia and reduces drug consumption after surgery by modulating these two pain perception mechanisms [21,109]. 

Regarding mental disorders, the interaction between bottom–up and top–down processes is related to the promotion of emotions [110,111]. Bottom–up perceptual mechanisms are related to the perceptual and affective stimuli processed by the basolateral amygdala [110]. Thus, anxiety disorders may be caused by hyperactivity in bottom–up processing mechanisms [112].

On the other hand, top–down mechanisms are related to cognitive interpretations carried out by the MPFC [110,112]. However, bottom–up perception mechanisms are sensitive to top–down influence, and greater activity in the limbic and sensory brain areas in response to a threat stimulus leads to the development and maintenance of anxiety states [111,112]. The interaction between brain areas related to top–down and bottom–up perception processes is promoted by theta oscillations, which synchronize limbic structures such as the amygdala and hippocampus with the MPFC [112].

TEAS modulation of top–down and bottom–up perception mechanisms likely synchronizes theta brain oscillations to promote a reduction in anxiety and improve the quality of life of cancer patients [113,114], as well as reducing pre- and postoperative anxiety in surgical patients [115,116].

In addition to these clinical fields, top–down and bottom–up neural mechanisms underlie distractibility behavior in adults with ADHD [117]. Adults with ADHD exhibited increased event-related potential (ERP) amplitudes in trials with low and high task difficulty compared to healthy controls, indicating impaired top–down and bottom–up processes modulated by the emotional content of the stimuli [117,118]. These results may explain the reduced functional activation of bottom–up and top–down networks found in adults with ADHD [119].

Recent systematic reviews and meta-analyses have demonstrated the limited effectiveness of acupuncture for ADHD, at least in part due to poor study quality [120,121]. On the other hand, TEAS has shown results in ADHD children, with treatment improving general symptoms, as well as promoting greater prefrontal area activation, as measured by functional near-infrared spectroscopy (fNIRS), thereby demonstrating the capacity of TEAS to modulate neural circuits in ADHD [122].

There are some potential limitations that must be considered when interpreting these results. First, despite the medium-to-large effect sizes found in this study that confirm the differences between stimulation periods and between TEAS groups, the diversity of the sample should be considered, such as the imbalance between females and males and the age variability of the participants. Furthermore, some study participants (more males than females) clearly preferred stronger stimulation, while others preferred it to be relatively gentle (more females than males) [123], so that stimulation amplitude may have been a confounding factor. Second, this crossover design was applied in healthy subjects, but, even so, supports future studies based on these current findings that could be applied to clinical populations. Third, although the routine clinical use of low-density EEG for source localization—as in this study, with only 19 channels—can lead to diminished precision in localization and blurring of the solution [124], sensor reduction has nonetheless become a topic of interest for researchers in an attempt to reduce system complexity, computation time, and system cost. It has been shown that, even with a reduced number of sensors, source localization can be performed with an acceptably low localization error and computation time [125]. Several recent studies on different clinical conditions, such as fibromyalgia, obsessive–compulsive disorder (OCD), tinnitus, autism spectrum disorder (ASD), and bipolar euthymia, have also used a low number of sensors for source estimation [126,127,128,129,130,131].

## 5. Conclusions

This study has revealed significant findings in the cortical modulation of the brain when using different TEAS frequencies, contributing new knowledge to the acupuncture field. Analysis demonstrates the possible neural mechanisms that relate to the modulation of the CNS promoted by peripheral electrical stimulation through a tactile stimulation effect involving both top–down and bottom–up factors. We observed that low TEAS frequencies of 2.5 and 10 pps modulated theta and alpha oscillations in brain areas related to emotional and attentional processes driven by external stimuli. In addition, the sham group demonstrated local neural synchronization of delta rhythms in the somatosensory areas of the brain likely related to expectation of stimuli at baseline. The authors hope that these findings will stimulate further research to evaluate these effects in real-world patients with regard to clinical outcomes such as analgesia and anxiety.

## Figures and Tables

**Table 1:** Demographic and clinical characteristics of the study sample. Values are presented as mean and standard deviation (SD) or relative frequencies (RFs) and percentage (n = 48).

Demographic Measures	Mean (SD) or RF (Percentage)
Age (years)	42.75 (17.27)
Sex (female/male)	30/18 (62.5%/37.5%)
Right-handed (Yes/No)	46/2 (95.8%/4.2%)
Caffeine consumption (Yes/No)	42/6 (87.5%/12.5%)
Alcohol consumption (Yes/No)	35/13 (72.9%/27.1%)
Nicotine consumption (Yes/No)	2/46 (4.2%/95.8%)
Past acupuncture treatment (Yes/No)	23/25 (47.9%/52.1%)
Past electroacupuncture treatment (Yes/No)	7/41 (14.6%/85.4%)
Past TENS treatment (Yes/No)	12/36 (25%/75%)
Medication use (Yes/No)	14/34 (29.2%/70.8%)
Medication Categories	
Antihypertensive	3/14
Contraceptive	2/14
Allergy	2/14
Hypothyroidism	2/14
Migraine	1/14

**Table 2:** Brain areas and the coordinates in which significantly voxel clusters were detected for the comparison performed between post-stimulation time (Slots 6, 7, and 8) and baseline (Slot 1) for the 2.5 pps, 10 pps, 80 pps, and sham TEAS groups. EEG cortical sources are based on MNI templates (n = 48).

*p*	t	Cluster	BA	MNI Coordinates			Freq.	TEAS Freq.	Comparison
				z	y	x		(GROUP)	
<0.01	4.42	Right Parahippocampal Gyrus	BA27	−5	−35	20	theta	2.5 pps	Post 1 (Slot 6) vs. Baseline (Slot 1)
<0.05	−4.2	Left Inferior Parietal Lobe	BA40	30	−35	−55	alpha	10 pps	
<0.05	−3.97	Left Postcentral Gyrus	BA03	55	−25	−40	delta	160 pps	
<0.01	3.97	Right Parahippocampal Gyrus	BA27	0	−35	15	theta	2.5 pps	Post 2 (Slot 7) vs. Baseline (Slot 1)
<0.01	−4.2	Left Postcentral Gyrus	BA02	30	−30	−40	alpha	10 pps	
<0.01	−4.35	Left Postcentral Gyrus	BA03	60	−30	−40	delta	160 pps	
<0.05	−4.07	Left Inferior Parietal Lobe	BA40	30	−40	−60	alpha	10 pps	Post 3 (Slot 8) vs. Baseline (Slot 1)

**Table 3:** Brain areas and the coordinates in which significantly voxel clusters were detected for the comparison performed between the 2.5 pps, 10 pps, and 80 pps TEAS groups and the sham TEAS group regarding the difference in post-stimulation time (Slots 6, 7, and 8) and baseline (Slot 1). EEG cortical sources are based on MNI templates (n = 48).

*p*	t	Cluster	BA	MNI Coordinates			Freq.	TEAS Freq.	Condition
				z	y	x			
<0.05	−4.31	Left Inferior Parietal Lobe	BA40	30	−35	−65	alpha	10 pps—Sham	Post 1—Baseline
<0.01	−4.42	Left Insula	BA13	20	−20	−35	alpha	10 pps—Sham	Post 3—Baseline

## Data Availability

The EEG data presented in this study will soon be freely available in the ongoing Human Brain Indices (HBI) reference database (https://www.hbimed.com) (accessed on 13 December 2022), and in Open Research Data Online (ORDO), The Open University’s searchable research data repository at https://ordo.open.ac.uk/ (accessed on 13 December 2022).

## Supplementary Materials

The following supporting information can be downloaded at: https://www.mdpi.com/article/10.3390/brainsci15030270/s1, Figure S1: Results of a PubMed search for “(TEAS OR TAES) AND transcutaneous”; Table S1: TEAS Neuroimaging studies (13) located in PubMed and listed in date order; Table S2: Head-to-head studies located in PubMed comparing single TEAS frequencies; Table S3: TEAS studies for different conditions found in Electroacupunctureknowledge.com (EAK) and PubMed.

## Abbreviations

AACP	Acupuncture Association of Chartered Physiotherapists
ACC	Anterior cingulate cortex
ADHD	Attention-deficit/hyperactivity disorder
AG	Angular gyrus
ASD	Autism spectrum disorder
BA	Brodmann’s Areas
BOLD	Blood oxygenation level-dependent
CCI	Chronic constriction injury
CNS	Central nervous system
DMN	Default mode network
DRG	Dorsal root ganglia
EA	Electroacupuncture
EC	Eyes-closed
ECG	Electrocardiogram;
ECI	Electro Cap International
EEG	Electroencephalography
EO	Eyes-open
ERP	Event-related potentials
ES	Electrical stimulation
fMRI	Functional magnetic resonance imaging
fNIRS	Functional near-infrared spectroscopy
HBI	Human Brain Indices
ICA	Independent Component Analysis
IFG	Inferior frontal gyrus
IPG	Inferior parietal gyrus
LFP	Local field potentials
LI4	Large intestine 4
MDD	Major depressive disorder
MNI	Montreal Neurological Institute
MPFC	Medial prefrontal cortex
MTG	Medial temporal gyrus
NITRC	Neuroimaging informatics tools resources collaboratory
NMES	Neuromuscular electrical stimulation
OCD	Obsessive–compulsive disorder
ORDO	Open Research Data Online
PAG	Periaqueductal gray
PCC	Posterior cingulate cortex
PES	Peripheral electrical stimulation
PL	Parietal lobe
PNS	Peripheral nervous system
POD	Postoperative delirium
PPC	Posterior parietal cortex
PPG	Photoplethysmography.
pps	Pulses per second
RF	Relative frequencies
RVM	Rostral ventral medulla
SI	Primary sensory area
SII	Secondary sensory area
SD	Standard deviation
sLORETA	Standardized low-resolution brain electromagnetic tomography
SMA	Supplementary motor area
SMG	Supramarginal gyrus
SMN	Sensorimotor network
SnPM	Statistical nonparametric mapping
TEAS	Transcutaneous electrical acupoint stimulation
TENS	Transcutaneous electrical nerve stimulation
TKA	Total knee arthroplasty
TMS	Transcranial magnetic stimulation
TPJ	Temporoparietal junction
VAS	Visual analog scale
