# Supplementary material for: Effect of Different Frequencies of Transcutaneous Electrical Acupoint Stimulation (TEAS) on EEG Source Localization in Healthy Volunteers: A Semi-Randomized, Placebo-Controlled, Crossover Study
Source: Brain Sci. 2025 Mar 3;15(3):270. doi: 10.3390/brainsci15030270 (PMC11940437; doi:10.3390/brainsci15030270)
Supplement: Supplementary file 1 [file brainsci-15-00270-s001.zip › brainsci-3399365-supplementary.pdf]

Transcutaneous electroacupuncture stimulation (TEAS): Neuroimaging and frequency – a brief narrative review

David Mayor

Visiting Research Fellow (Physiotherapy),
Department of Allied Health Professions and Midwifery,
School of Health and Social Work,
University of Hertfordshire, UK

Background

Transcutaneous electroacupuncture stimulation (TEAS), also known as 'transcutaneous electrical acupoint stimulation' or even 'transcutaneous acupoint electrical stimulation' (TAES), is a variant of electroacupuncture (EA) that does not make use of needles to transmit current to the body, but surface electrodes. It can thus be seen as a non-invasive fusion of the Western method of transcutaneous electrical nerve stimulation (TENS) with Eastern acupuncture. The creation of TENS in the 1960s is usually credited to the American C Norman Shealy (1932-2024), and the first patent application for TENS (as a treatment specifically for pain) was filed in the US in 1972 [Anon nd]. In China, electroacupuncture was first used in the 1930s, and 'acupuncture analgesia' in the 1950s. Both TENS and EA have nineteenth century historical antecedents [Mayor 2007].

With the intention of locating the main, but not necessarily all, published studies on TEAS, only two online resources were used: PubMed, from the US National Library of Medicine [https://pubmed.ncbi.nlm.nih.gov/] and Electroacupunctureknowledge.com, a companion to Elsevier's textbook *Electroacupuncture: a practical manual and resource* [Mayor 2007]. For the latter, paper copies of the original studies were sourced from the British Library in London, the Needham Research Institute in Cambridge (UK) and other libraries. Assistants were co-opted to translate from Chinese and Russian/Ukrainian/Belarusian sources when necessary.

There are currently (7 February 2025) 8,272 electroacupuncture studies listed in PubMed, some dated as far back as 1960, with 344 on "transcutaneous electrical acupoint stimulation" and 53 on "transcutaneous acupoint electrical stimulation", all published between 1994 and 2025. Electroacupunctureknowledge.com includes data entered no later than 2005 and extracted from over 8,000 clinical studies on electroacupuncture and related methods. Of these, 151 were on TEAS – although there were duplicate entries for some studies where TEAS was used for several different conditions. The earliest TEAS study included in the Electroacupunctureknowledge database was published in 1976, the most recent is from 2003. As a comparison, in PubMed only 8 studies on TEAS (or TAES) were indexed for the years up to and including 2003 (**Figure 1**).

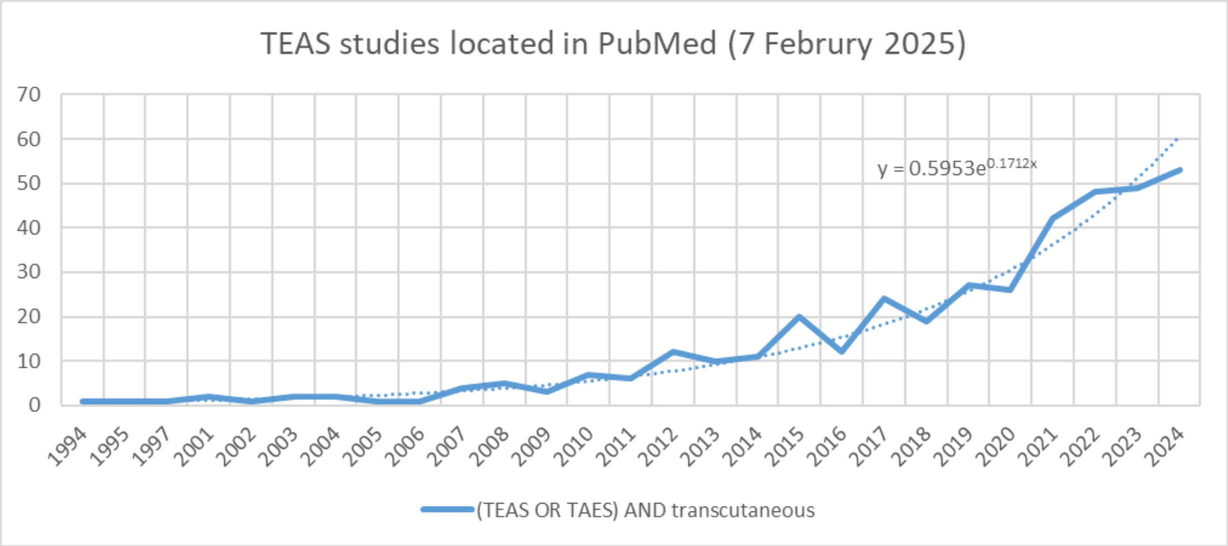

Figure S1. Results of a PubMed search for "(TEAS OR TAES) AND transcutaneous" up until and including 2024 (search conducted on 7 February 2025). The term 'transcutaneous' was included in the search string to filter out studies on herbal teas. As with most searches, numbers of 'hits' increase exponentially over time, as shown in Figure 1. Here the exponent is 0.1712, indicating quite rapid growth.

With limited time available, more restricted PubMed searches were then conducted to keep this review within manageable bounds – using the strings "(TEAS OR TAES) AND transcutaneous AND (Hz OR frequenc*)" (112 results, 1997-2004, exponent 0.1465) and "Transcutaneous AND (TEAS OR TAES) AND (EEG OR fMRI OR MEG OR fNIRS OR PET" (13 results, 2012-2023, exponent only 0.002).

Selected findings from these two searches are presented below in tabular form.

History

TEAS in the West

The earliest TEAS studies date back to the 1970s. The American authors of the first (1976) study included in Electroacupunctureknowledge.com argued that both EA and TEAS may have more effect on the affective dimension of pain (as measured on a scale of subjective unpleasantness) than its sensory dimension (intensity) [Goldberger and Tursky 1976].

The following year, a Scandinavian (Danish/Swedish) group published an experimental study in which TEAS was applied at the acupuncture point LI4 (*hegu*) on the hand, located on the skin between the thumb and forefinger [Sjölund et al. 1977]. The same point (with return electrode on the ulnar border of the hand) was used in several studies a few years later by Birger Kaada (1918-2000), a Norwegian neurophysiologist [Kaada & Eielsen 1983b; Kaada et al. 1990].

Kaada was a proponent of 'acupuncture-like TENS' (ALTENS, low-frequency bursts or trains of high-frequency stimulation – typically 2-4 Hz bursts of 80-100 Hz). In several studies, he found that LF TEAS at LI4 resulted in widespread, if superficial, vasodilation

[Kaada & Eielsen 1983a-c]. He considered this, in part, to be the result of sympatho-inhibition [Kaada & Helle 1984]. Others, more sceptical, suggested that apparent changes in blood flow with motor level low-frequency stimulation may in fact be the result of acclimatisation [Sherry et al. 2001].

Nonetheless, autonomic changes in response to TEAS remain a topic of interest, with an increasing number of studies now including measures of heart rate variability (HRV) to assess parasympathetic and sympathetic activity [e.g., Cai et al. 2024; Cong et al. 2024; Jesus et al. 2021; Li et al. 2016; Li et al. 2019; Low et al.2024; Moreira et al. 2019; Zhu et al. 2022]. Some of these studies – like our own – have used both HRV *and* neuroimaging methods concurrently.

An alternative definition of ALTENS – as simply the use of TENS at acupuncture points, or TEAS in other words – has been advocated by those using the Canadian Codetron device developed in the 1980s by Brue Pomeranz, Norman Salansky and others [Pomeranz & Niznick 1987; Salansky et al. 1998]. To overcome the habituation that can result from using 'conventional', repetitive high-frequency (HF) TENS at low intensity, the acupuncture points are stimulated in random order using trains of low-frequency (LF) pulses that produce a stronger sensation [Wong et al. 2003].

TEAS in China – focusing on the work of Han Jisheng

On the other side of the world, Han Jisheng, a member of the original 'Research Group of Acupuncture Anesthesia' in Peking (and later director of the Neuroscience Research Institute at Beijing University), had already started to publish papers on acupuncture in 1973 (Research Group of Acupuncture Anesthesia 1973). With his co-authors, he noted that pain threshold was increased by manual acupuncture at LI4 or ST36 (*zusanli*), a point on the leg. Analgesia was stronger if both LI4 and ST36 were used together. Furthermore, both the sensation evoked by needling LI4 and the analgesia that resulted were identical to those obtained by needling a *non*-acupuncture point between the second and third metacarpals, so a little distance from the traditional location of LI4. In other words, "the specificity of acupuncture points is relative" [Research Group of Acupuncture Anesthesia 1973].

Despite this, Han – and many other hard-nosed scientific researchers – have continued to use the traditional acupuncture points LI4 and ST36 most often in their investigations into the effects of TEAS, as well as other traditional acupuncture points such as P6 and SJ5, Sp6 and Liv3, and so on.

Sham TEAS

For comparison with the 'verum' treatment, active TEAS is sometimes applied to sham (non-traditional) points [e.g., Chung et al. 2014; So et al. 2007]; alternatively, zero- or low-amplitude, clinically ineffective TEAS may be applied at the same traditional points as the active treatment [Gao et al. 2022; Meade et al. 2010; Mi et al. 2018; Song et al. 2020; Zhou et al. 2015]. Sometimes both strategies are invoked, inactive TEAS being applied to non-traditional points [Zhou et al. 2018]. All these have been called 'sham' TEAS. In our

own study, as we have described, we used the second approach – low-amplitude, clinically ineffective TEAS, albeit at a high frequency (160 pps), applied at LI4 itself.

Han even developed a 'Mock HANS' device for use in blinded research studies, with output fixed at 5mA, just above the sensory threshold (3 or 4mA), and intermittent (10 seconds on, 20 seconds off) [Jiang et al. 2012].

Han (born in 1928) has continued to investigate the effects of TEAS (and electroacupuncture, EA) on the neurochemistry of acupuncture-related methods of stimulation well into his 90s. The earliest of his 185 studies listed in PubMed (on acupuncture tolerance and anti-opiates) dates back to 1979 [Han et al.], the most recent, on brain structural connectivity in autism, to 2023 [Jiang et al. 2023]. Two volumes of his collected papers on *The Neurochemical Basis of Pain Relief by Acupuncture* are available in English (Han 1987, Han 1998).

One of Han's major contributions to the neurophysiology of acupuncture was to disentangle the effects of low- and high-frequency stimulation, whether EA or TEAS. To summarise, "it was concluded that low-frequency (2 Hz) EAA [electroacupuncture analgesia] is induced by the activation of mu- and delta-opioid receptors via the release of enkephalin, beta-endorphin, and endomorphin in supraspinal CNS regions, whereas the effects of high-frequency (100 Hz) EAA involve the actions of dynorphin on kappa opioid receptors in the spinal cord. A combination of the two frequencies produces a simultaneous release of all four opioid peptides, resulting in a maximal therapeutic effect" [Bioregulatory Medicine Institute, https://www.brmi.online/ji-sheng-han]. This 'dense-disperse' (perhaps more accurately, 'disperse-dense') stimulation has the added advantage that varying the frequency can, to some extent, counter the effects of habituation to an unchanging single frequency of stimulation [DeSantana et al. 2008]. Numerous Chinese studies have used the HANS 100A, HANS 200A or other TEAS devices based on Han's research into the effects of stimulation frequency, usually applied at a ''maximal but comfortable level".

TEAS and neuroimaging

As the technology has become more available, more recent studies have often used neuroimaging methods, in addition to or instead of neurochemical ones. Using the search string "(TEAS OR TAES OR electroacupuncture OR electro-acupuncture) AND [acronym for imaging method]" in PubMed, it appears that functional magnetic resonance imaging (fMRI) has been the most commonly used method (103 'hits' from 2001-2024, but only 6 of them for TEAS/TAES), followed by the less costly electroencephalography (EEG) (52 hits, 1975-2024, but only 8 since 2001 and only 8 of the 52 for TEAS/TAES). Magnetoencephalography (MEG), the most costly method, was very little used (3 hits, 2008-2024, with none of them being for TEAS/TAES). Only one study was located for functional near-infrared spectroscopy (fNIRS), and one for positron emission tomography (PET). Given that acupuncture-based research is relatively underfunded, the predominance of EEG before the new millennium is not surprising. Results are summarised in **Table S1** below.

Functional connectivity in the brain is another area of rapidly increasing interest. Using the string "(TEAS OR TAES OR electroacupuncture OR electro-acupuncture) AND "functional connectivity"," 60 studies published between 2009 and 2024 were located in PubMed (although only 3 of them for TEAS/TAES). These were all fMRI studies; none involved EEG.

Han Jisheng has himself co-authored a number of studies on EEG [Qi et al. 2003 [EEG]; Zhang et al. 2003 [EEG]; Zuo et al. 2007 [EEG]; Wang et al. 2013 [EEG]], fMRI [Zhang et al. 2003 [fMRI]; Zhang et al. 2003 [fMRI]; Zhang et al. 2004 [fMRI]; Jiang et al. 2012 [fMRI]; Jiang et al. 2013 [fMRI]; Zhang et al. 2013 [fMRI]] and PET [Xiang et al. 2014 [PET]], as well as on functional and even *structural* brain connectivity in autism (the latter using diffusion tensor imaging DTI) [Shou et al. 2017; Jiang et al. 2023]. Several of these focus on the effects of TEAS on pain [Zhang et al. 2003; Zhang et al. 2003; Zhang et al. 2003], and one on the particular brain regions activated or deactivated by "the stimulation of acupoints" [Zhang et al. 2004]. Han's 2012 fMRI study [Jiang et al. 2012] also investigated the effects of TEAS on functional connectivity. In a further fMRI study the same year, his research group noted the activating and deactivating effects of high-frequency EA at LI4 on blood oxygenation level and cerebral blood flow in different regions of the brain [Zhang et al. 2012].

Table S1. TEAS Neuroimaging studies (13) located in PubMed and listed in date order, showing first author and date (in **bold**, studies for which Han Jisheng was a co-author), participants and controls, neuroimaging method and stimulation parameters, with some main findings.

Paper	Participants/ Control	Methods	Main finding/s
**Jiang 2012**	Healthy/ MTEAS	fMRI<br>2 Hz<br>L LI4-P6<br>30 min	Global CBF decreased, with significant regional decrease of regional CBF in SI, insula, STG, MOG and IFG. Functional connectivity stronger and more extended connectivity in both DMN and SMN after long-duration TEAS
Penetar 2012	Drug dependent / 2 min sensory	EEG, ERP<br>2/100 Hz<br>L P6-SJ5<br>R LI4-P8<br>30 min	Cocaine cravings/use not changed, but significantly less self-reported anxiety, tension/agitation* & irritability*, with improved concentration (* also for marihuana)
**Zhang 2013**	Healthy/ minimal MTEAS	fMRI<br>2 Hz<br>L LI4-P8<br>30 min	Decreased local efficiency, changed nodal efficiency in frontal gyrus, OFC [paralimbic], ACG, hippocampal gyrus [limbic] {ACG & OFC are deactivated by acupuncture}; long-duration TEAS modulates short range connections in functional networks and limbic system
**Jiang 2013**	Healthy/ MA,EA, tapping	fMRI<br>2 Hz<br>L ST36 & proximal<br>1 & 5 min	More secure and spatially extended connectivity of DMN with MA and EA; TEAS specifically increased functional connectivity in SMN; more extensive deactivation with EA than MA or TEAS

Wang 2014	Sinusotomy patients/ Same points, no stimulation	EEG (BIS)<br>2/10 Hz<br>Bilat LI4, P6, ST36<br>30 min	TEAS significantly reduced intra-operative remifentanil and alleviated postoperative side-effects
**Xiang 2014**	Monkeys/ Same points, no stimulation	PET<br>2 or 100 Hz<br>LI4, P8<br>30 min	2 Hz but not 100 Hz TEAS evoked significant increase in MOR<br>binding potential in ACG, caudate nucleus, putamen, temporal lobe, SI and amygdala compared with 0 Hz TEAS (all regions related to pain and sensory processes); post-TEAS, effect remained in ACC and temporal lobe
**Jiang 2014**	Healthy/ MTEAS	fMRI<br>2 or 100 Hz<br>L LI4-P8<br>30 min	analgesic effect started at 20 min; in both TEAS groups, trend in regional CBF of early activation with later inhibition; positive correlation between analgesia and regional CBF change in<br>anterior insula (early stage), with negative relationship in parahippocampal gyrus (later stage). TEAS analgesia specifically<br>associated with DMN and other cortical regions in 2-Hz TEAS group, ventral striatum and dorsal ACG in 100-Hz TEAS group.<br>Mechanisms of LF and HF TEAS analgesia are distinct and partially overlap
Kong 2015	Bell's palsy/ Sham point 2 cm lateral to LI4	fMRI<br>5 Hz<br>contralateral LI4, ipsilateral ST6<br>3 min	Brain regions activated more by TEAS at real than at sham point; brain regions activated by LI4 [L MTG, R SMG, L ITG, L posterior cerebellum] and ST6 TEAS were broadly overlapping and adjacent; "results provide supplementary neuroimaging evidence for … acupoint specificity"
Liu 2016	Pituitary adenoma resection under propofol sedation	EEG<br>2/100 Hz<br>L LI4-SJ5, ST36-GB40<br>2 x 2 min	EEG alpha and beta band power increased in light propofol sedation, but reduced in delta and beta bands in deep propofol sedation; synchronization (coherence) between channels enhanced in LF bands, declined in HF bands, whether sedation light or deep; TEAS may enhance sedative effect of low concentration propofol but reduce that of high concentration propofol
Yan 2022	Healthy/ at rest	EEG (BIS)<br>2/10 Hz<br>Bilat ST36, HT7, SP6<br>30 min	TEAS-induced sedation accompanied by reduced alpha power and increased delta power; Permutation entropy lower during<br>TEAS session, suggesting reduced EEG complexity; significantly reduced global strength of functional connection (wPLI) in delta and alpha during TEAS; local and global efficiency of brain functional network lower during TEAS session, but characteristic<br>path length higher; BIS significantly reduced during TEAS (max reduction in BIS after 15 min)

Zhuo 2023	ADHD/ Same points, no stimulation	fNIRS 2/10 Hz Bilat KI3, LIV3; DU20 20 min	Clinical Global Impression-Severity of Illness (CGI-S) score and mean cerebral oxygenated hemoglobin within the prefrontal cortex improved more in 4 weeks with TEAS than sham TEAS
Wang 2023	Healthy/ at rest, or non-point near elbow flexure	EEG (sLORETA) 2 Hz R P7 2 x 2 min	phase-locking value (PLV), and complex network: mainly activated frontal lobe and temporal lobe including prefrontal cortex (BA10), insular lobe (BA13), temporal gyrus (BA22), anterior cingulate cortex (BA32), temporal pole (BA38), dorsolateral prefrontal cortex (BA46), and inferior frontal cortex (BA47) – all closely linked to cognition and emotion; degrees of node in frontal, temporal, and whole brain increased; clustering coefficient in frontal, temporal, and whole brain all increased relative to resting state
Lu 2023	Healthy/ Same points, no stimulation	fMRI 2/10 Hz Bilat HT7-P4 30 min	compared with controls, ALFF in TEAS group decreased in L thalamus, R putamen and midbrain, increasing in L OFC. More FC between thalamus and insula, MCC, SI, amygdala and putamen after TEAS than non-stimulation. Thalamus ALFF positively correlated with BIS in both groups.

ACG: anterior cingulate gyrus; ALFF: amplitude of low-frequency fluctuations; BIS: Bispectral index; CBF: cerebral blood flow; DMN: default mode network; EA: electroacupuncture; FC: functional connectivity; fNIRS: functional near-infrared spectroscopy; HF: high-frequency; IFG: inferior frontal gyrus; ; ITG: inferior temporal gyrus; LF: low-frequency; MA: manual acupuncture; MCC: middle cingulate cortex; MOG: middle occipital gyrus; MOR: μ-opioid receptor; MTEAS: mock TEAS, here 2 Hz, < 5 mA (i.e., sensory, not motor, level), intermittent; MTG: middle temporal gyrus; OFC: orbitofrontal cortex; PET: positron emission tomography: SI: somatosensory cortex; SMG: supramarginal gyrus; SMN: sensorimotor network; STG: superior temporal gyrus; wPLI: weighted phase lag index (wPLI).

TEAS and frequency

There are relatively few studies located via either PubMed or Electroacupunctureknowledge.com that compare the effects of single stimulation frequencies 'head-to-head' (rather than combinations of frequencies, such as alternating high low and high frequencies, as in 'dense-disperse' stimulation). The 17 studies located in PubMed are shown in **Table S2** below.

Table S2. Head-to-head studies located in PubMed comparing single TEAS frequencies (Han's studies in **bold**).

Author 1	Frequencies	Points	Main finding/s
**Han 1994**	2 v 100 Hz	LI4-palmar thenar ST36-BL57	Spinal spasticity: HF TEAS may help to relax spasticitybriefly, but LF TEAS may not.
Attele 2003	4 v 32 Hz	LI4-P6 15 min	Healthy: no significant difference between pain scores at 4 Hz and 32 Hz.
**Xiang 2014**	2 v 100 Hz	LI4-P8	Monkeys; 2 Hz (not 100 Hz) TEAS evoked a significant increase in MOR binding potential in anterior cingulate cortex, caudate nucleus, putamen, temporal lobe, SI and amygdala compared with 0 Hz TEAS; effect remained after TEAS in anterior cingulate cortex and temporal lobe.
Zhao 2015	2 v 100 Hz	LI4-LU10 ST36-BL57	Muscle spasticity; compared with 2 Hz or sham TEAS, 100 Hz TEAS decreased wrist spasticity at weeks 2, 3, and 4 of treatment and 1 month after treatment.
Yang 2016	2 v 15 v 100 Hz	St36, P6 v non-point	Rats; TEAS preconditioning, especially at moderate and high frequency, mitigates short-term adverse morphological changes induced by overload training.
Huang 2017	2/100, 2, and 100 Hz	ipsilateral P6, LI4, LI11, LU7	Surgical lobectomy; in 100 Hz group incidence of postoperative nausea and vomiting (PONV) lower than control group; intraoperative opioid consumption lowest in 2/100 Hz group.
Yu 2017	2 v 100 Hz	GB34, ST36, DU26	Rat model of neuropathic pain; MOR expression in L3-L5 dorsal root ganglion significantly increased by LF (not HF) TEAS.
Jin 2017	2 v 100 Hz	Bilat BL23, L ST36, Ren4	Asthenozoospermia; both 2 Hz and 100 Hz TEAS are effective for treatment of, improving sperm motility and vitality.
Qu 2017	2 v 100 v 2/100 Hz v control	8 points	In Vitro Fertilization (IVF); no difference in numbers of metaphase II oocytes, normally fertilized zygotes, early cleavage embryos or good quality embryos, but CPR, IR, LBR and follicular fluid NPY of 2/100Hz group significantly higher than other groups.
Lu 2019	2 v 100 Hz v 2/100 Hz	? & P6	PONV; 2/100 Hz TEAS combined with wristband pressing at P6 is more effective for PONV. 2/100 Hz TEAS and 100 Hz TEAS combined with P6 wristband have postoperative analgesic effect, better with 2/100 Hz TEAS.
Yu 2019	2 v 100 Hz v sham	BL23, ST36, Ren1, Ren4	Male infertility; 2 Hz TEAS improved sperm count and motility in patients with abnormal semen parameters, associated with increases in seminal plasma zinc, NAG and fructose, upregulation of CIB1 and downregulation of CDK1.

Gong 2021	2 v 100 Hz	Various	Male infertility; Neither 2 Hz nor 100 Hz TEAS had any significant effect on sperm concentration, while 100 Hz TEAS markedly improved percentage of grade a sperm compared with 2Hz TEAS. In comparison with blank control, neither 2 Hz nor 100 Hz TEAS significantly affected percentage of grade a + b sperm or total sperm motility. Conclusions: effect of TEAS on pregnancy outcome is not yet clear.
Jin 2021	2 v 100 Hz	BL23, ST36, Ren1, Ren4	Asthenozoospermia; both 2 Hz and 100 Hz TEAS significantly improved sperm motility and viability; effective rate of 100 Hz (93.75%) was higher than for 2 Hz (65.85%), but no significant alteration observed in CatSper1 and CatSper3 protein abundance in sperm of 100 Hz-TEAS treated patients, whereas (a significant increase in abundance of CatSper protein with 2 Hz.
Ma 2023	2 v 10 Hz v usual care [Bang et al. compared 3, 10, 30 & 300 Hz for median nerve stimulation]	LI11, LI4; P6, P4; ST36, LIV3	Hypertension (study protocol): "To our knowledge, apart from evaluating efficacy and safety of TEAS for hypertension, few studies have focused on the **optimal stimulus parameters** for TEAS treatment"; LF stimulation reduces BP in animal experiments and clinical trials; EA frequency for hypertension is mostly 2 Hz, proven to **inhibit sympathetic outflow** and induce dilation of systemic arteries, resulting in a suppressive effect on BP. Bang et al. found 10 Hz transcutaneous median nerve stimulation most comfortable, and effective, for lowering SBP.
Liu 2024	LF v HF v control	P6	Hypotension after spinal analgesia; Lower incidence of hypotension in HF group by 30 min after spinal analgesia than in control and LF groups; patients in HF group showed lower incidence of dizziness, dyspnea and chest congestion than in other 2 groups.
Ma 2024	2 Hz v 10 Hz	Bilat LI11, LI4, P6, P4, ST36, LIV3	Hypertension; 10 Hz provided a more significant decrease in SBP than 2 Hz; both TEAS groups exhibited more reductions in DBP, MAP, HR, HRV LF/HF ratio, VLF), LFnu, and an increase in HFnu than control. No differences observed among groups in LF, HF, TP, VLF or SF-12.
Yang 2024	50 Hz (250 µs) v 2 Hz (50 µs)	Bilat ST6, SJ17	Xerostomia; both groups showed significant improvement in dry mouth after treatment (saliva flow greater with 50 Hz TEAS ($p < 0.0001$). Patients less likely to perceive they were in contrast group in comparison with a study that used no stimulation.

BP: blood pressure; CDK1: cyclin-dependent kinase 1; CIB1: calcium and integrin-binding protein 1; CPR: clinical pregnancy rate; DBP: diastolic blood pressure; HF: high frequency;

HR: heart rate; HRV: heart rate variability; IR: implantation rate; IVF: in vitro fertilization; LBR: live birth rate LF: low frequency; MAP: mean arterial pressure; MOR: µ-opioid receptor; MTEAS: Mock TEAS; NPY: neuropeptide Y; nu: normalized units; PONV: postoperative nausea and vomiting; SBP: systolic blood pressure; SF-12: 12-item Short Form Health Survey; SI: somatosensory cortex; SpO_2: oxygen saturation; TP: total power; VLF: very low frequency.

Clearly, for such a heterogeneous collection of studies and conditions, there is no clear 'winner'. In some situations, HF stimulation gives better results, in others LF TEAS. Mid-range (10 Hz or 15 Hz) is less commonly the 'best' frequency to use, although this may be the case for systolic blood pressure [Ma et al. 2024], although HRV measures did not in their study differ with stimulation frequency.

Research into the effects of different frequencies of stimulation is very much ongoing, with numerous gaps in the evidence that remain to be filled. As many of the systematic reviews located via PubMed repeat, almost as a mantra, the quality of current evidence is limited, and there is a need for high-quality randomised controlled trials to verify the tentative results already obtained [Gong et al. 2021; Tu et al. 2021; Yang et al. 2022; Zhu et al. 2022; Ge et al. 2023; Wei et al. 2023; Wang et al. 2024]. It may be of interest to note that our own HRV findings suggest that 10 pps TEAS may be experienced as less stressful during and after stimulation than TEAS at the other frequencies used [Mayor et al. 2019].

The uses of TEAS

Although TENS was used originally as a method of pain control, following the publication of the 'gate control' theory of pain by Melzack and Wall in 1965 [Melzack & Wall 1965], it would be oversimplistic to state that acupuncture, TEAS or EA are only useful for painful or musculoskeletal conditions.

Like acupuncture itself, as can be seen by referring to any textbook [e.g., Li 2007], TEAS has in fact been used for a wide range of conditions, a veritable 'kitchen sink of them', not simply those involving pain – or, indeed, anxiety, although there are 164 studies in PubMed on "(TEAS OR TAES) AND transcutaneous AND (pain* OR analg*)", indexed between 1994 and 2024, and 51 studies on "(TEAS OR TAES) AND transcutaneous AND (anxi* OR stress* OR relax*)", indexed between 2007 and 2024.

In the online database at Electroacupunctureknowledge.com, for example, two or more entries on TEAS or SSP (silver spike point stimulation, a Japanese variant of TEAS) can be found for a variety of conditions, as shown in the following Table. By way of comparison, numbers of hits in PubMed using the same terms in a basic search are also shown. However, as some of the EAK terms were not recognised by PubMed, the resulting study counts are not so useful as for EAK. Although alternative strategies were sometimes used in an attempt to widen the PubMed searches, lack of time has not allowed this to be done consistently and completely. Results are shown in **Table S3**.

Table S3. TEAS studies for different conditions found in Electroacupunctureknowledge.com (EAK) and PubMed, located using the Electroacupunctureknowledge categories to guide searches. Note that some studies in both EAK and PubMed covered the use of TEAS for

several different conditions, so that total numbers may be artificially inflated. Only conditions with 2 or more entries are included for EAK, whereas all conditions with 1 entry or more are included for PubMed.

Condition	*n* studies (EAK)	Date range	*n* studies (PubMed)	Date range
Analgesia – dental	12	1976-1991	n/a	
Analgesia – brain	3	1994-2001	30	1994-2003
Analgesia – eye	12	1978-1986	n/a	
Analgesia – neck/throat	6	1983-2001	6	2007-2022
Analgesia – gall bladder	3	1982-1991	n/a	
Addiction – nicotine	2	1988-1990	2	2011-2022
Addiction – opiates	8	1994-2001	2	2010-2021
Appetite control	4	1989-2003	5	2008-2022
Hypertension	6	1981-2000	16	2008-2024
Spasticity	3	1981-1994	3	2015-2023
Spinal injury	4	1993-2003	1	2014
PONV	11	1982-2002	70	2001-2024
CINV	7	1991-2003	11	1997-2024
Morning sickness	3	1991-2001	6	2003-2024
Motion sickness	2	1992-1995	n/a	
Other nausea/vomiting	2	1997-2003	n/a	
Oesophageal conditions	5	1983-1996	2	2020-2023
Abdominal pain	4	1986-1992	18	1997-2023
Gallstones	4	1987-1998	n/a	
Incontinence (neurol.)	4	1982-1984	n/a	
Urinary retention	2	1997-2000	5	2021-2024
Urinary tract stones	4	1976-2000	n/a	
Erectile dysfunction	4	1986-2000	n/a	
Migraine	8	1980-2002	n/a	
Tension-type headache	3	1980-1992	n/a	
TMJ disorder	9	1981-1998	n/a	
Trigeminal neuralgia	5	1982-2002	n/a	
Jaw problems	2	1985-1991	1	2024
Cancer	4	1993-1997	32	1994-2024
Cancer pain	6	1979-1997	58 [OE]	1994-2024
Radio/ chemotherapy	2	1991-2001	3 + 43 [OE]	2007-2024
Cervical spine disorders	9	1975-1997	n/a	
… nerve compression	3	1975-1990	n/a	
… soft tissue	2	1980-1986	n/a	

Shoulder girdle	11	1975-2000	n/a	
Wrist and hand	4	1981-1990	n/a	
LBP (general)	3	1980-1993	1	2007
LBP + nerve entrapment	3	1980-1990	n/a	
Chronic back pain	21	1974-1992	2	2007-2022
… + nerve entrapment	4	1974-1982	n/a	
Acute back pain	6	1983-1994	1	2007
… + nerve entrapment	2	1983-1984	n/a	
Sciatica	5	1986-2001	n/a	
Hip	4	1989-1992	3	2014-2024
Knee	12	1980-1994	13 [OE]	2007-2024
Arthritis (general)	4	1977-2002	n/a	
Osteoarthritis	9	1983-1994	1	2019
Rheumatoid arthritis	2	1981-1989	n/a	
Fibromyalgia/myofascial	7	1984-1997	1	2019
Soft tissue injury	5	1980-2000	1	2019
Postherpetic neuralgia	2	1979-1980	n/a	
Sensory nerve injury	2	1975-1992	n/a	
Phantom limb pain	5	1975-1989	n/a	
Peripheral neuropathy	3	1986-1999	2017-2024	2017-2024
CRPD	2	1975-1983	3 [OE]	
Central pain	2	1975-1982	2	2020-2023
Induction of labour	3	1988-1994	n/a	
Pain relief in labour	4	1980-2001	4	2020-2023
Dysmenorrhea, PMS	6	1985-1992	1	2024
Chronic pain	10	1979-1992	10	2007-2023
Mixed pain	10	1976-1997	n/a	
Facial paralysis	8	1986-2001	n/a	
Hysterectomy	2	1996-1998	n/a	
Gynecological surgery	3	1997-1999	9	2020-2023
Thoracic surgery	3	1992-1993	21 [OE?]	2012-2024
Abdominal surgery	2	1980-1993	21 [OE?]	1991-2024
Phobia	2	1984-1991	n/a	
Stress and relaxation	2	1989-1999	36 [OE]	2007-2024
Asthma	4	1977-1998	n/a	
Retinal disorders	2	1983-2003	n/a	
Myopia	2	1996-2002	n/a	
Deafness/hyperacusis	2	1992	n/a	
Tinnitus	3	1985-1992	n/a	
Rhinitis	2	1989-1990	n/a	
SUM	356	1976-2003	398	1991-2024

CINV: chemotherapy-induced nausea and vomiting; CRPD: Complex regional pain disorder; EAK: Electroacupunctureknowledge.com; LBP: low back pain; n/a: no relevant studies located using simple keyword searching; OE: an overestimate; PMS: premenstrual syndrome; PONV: Post-operative nausea and vomiting; TMJ: temporo-mandibular joint.

Discussion

This file of ‘Supplementary Material’ was created in response to comments by three anonymous academic reviewers on our paper about the effect of different frequencies of TEAS on EEG source localization in healthy volunteers. We trust that we have now addressed their concerns adequately.

We would like to point out that the EEG data for this study were collected almost a decade ago, in 2015-16, so that studies published since then were not part of our thinking when our own was being designed. Indeed, the groundwork for our research was already laid down in the late 1990s, and presented at a 2001 conference when the present author suggested that the central effects of peripheral stimulation like TEAS might be the result of neural ‘entrainment’ or ‘resonance’ [Mayor 2001], as already proposed by Salansky, among others [Salansky et al. 1998]. Our focus, therefore, has always been on the effects of single stimulation frequencies on the EEG, not on the effects of combinations of frequency (as in ‘dense-disperse’ TEAS), and not on the conditions treated.

Our narrative review demonstrates a number of important points and gaps in the literature.

From **Table S**1, it appears that EEG and fMRI have been the neuroimaging methods most commonly used in TEAS studies, but only two neuroimaging studies have compared the effects of different single frequencies of TEAS: Xiang et al. 2014 (PET) and Jiang et al. 2014 (fMRI). Neither of these is an EEG study.

In the 2015 fMRI study on Bell’s palsy by Kong et al., the authors found significant differences in the brain regions activated by TEAS at LI4 and a sham point 2 cm lateral to LI4. For them, this finding “shows acupoint specificity”. Other dyed-in-the-wool scientific researchers on TEAS like Han Jisheng continue to use LI4 in their work, rather than randomly selecting non-points for their neuroimaging (or other) studies, even though they might conclude, as Han did in 1994, that “the specificity of acupuncture points is relative”.

Only two studies were located that used ‘low-resolution electromagnetic tomography’ (sLORETA) [Wang et al. 2023; Zarei et al. 2022], supporting the need for further studies on TEAS and sLORETA, as in the present study. Furthermore, only one study was located on how TEAS may affect (permutation) entropy of the EEG signal [Yan et al. 2022]. Given the present authors’ interest in complexity and entropy in physiological signals [Mayor et al. 2021, 2023], this may be a fruitful avenue to explore in future.

There also appears to be scope for new research into the effects of different frequencies of TEAS on the EEG, particularly in conjunction with HRV analysis. Do low frequencies of stimulation relax (and enhance EEG theta, for instance, as well as HRV HF power) and high frequencies stimulate (and enhance EEG gamma and higher mental

processes, for instance, as well as HRV LF power)? Such binary/dualistic questions may appear naïve and even dated [Equinox Group c. 1988; Salansky et al. 1998], but that does not mean they are not worth asking any more [Ebrahimian et al. 2018; Xu et al. 2020; Zarei et al. 2022]. Research is a cyclical process.

References

1. Anon. nd. Transcutaneous stimulator and stimulation method. https://patents.google.com/patent/US3817254.
2. Attele AS, Mehendale S, Guan X, Dey L, Yuan CS. Analgesic effects of different acupoint stimulation frequencies in humans. Am J Chin Med. 2003;31(1):157-62. doi: 10.1142/S0192415X03000795. PMID: 12723766.
3. Cai YC, Lin YL, Yin SJ, Ding Y, Wu W, Mo KL, Shi JD, Song HJ. Effect of transcutaneous electrical acupoint stimulation based on the theory of "*qi* ascending and descending movement" on autonomic nervous system and gastrointestinal function in patients after general anesthesia laparoscopic cholecystectomy. Zhen Ci Yan Jiu. 2024 Mar 25;49(3):283-288. English, Chinese. doi: 10.13702/j.1000-0607.20221402. PMID: 38500325.
4. Chung YC, Chien HC, Chen HH, Yeh ML. Acupoint stimulation to improve analgesia quality for lumbar spine surgical patients. Pain Manag Nurs. 2014 Dec;15(4):738-47. doi: 10.1016/j.pmn.2013.07.010. Epub 2013 Oct 19. PMID: 24144572.
5. Cong L, Yu X, Huang M, Sun J, Lv H, Zhang T, Dang W, Teng C, Xiong K, Ma J, Hu W, Wang J, Cheng S. Enhancing emotion regulation: investigating the efficacy of transcutaneous electrical acupoint stimulation at PC6 in reducing fear of heights. Front Psychol. 2024 Apr 3;15:1371014. doi: 10.3389/fpsyg.2024.1371014. PMID: 38633874; PMCID: PMC11021653.
6. Desantana JM, Santana-Filho VJ, Sluka KA. Modulation between high- and low-frequency transcutaneous electric nerve stimulation delays the development of analgesic tolerance in arthritic rats. Arch Phys Med Rehabil. 2008 Apr;89(4):754-60. doi: 10.1016/j.apmr.2007.11.027. PMID: 18374009; PMCID: PMC2744433.
7. Ebrahimian M, Razeghi M, Zamani A, Bagheri Z, Rastegar K, Motealleh A. Does High Frequency Transcutaneous Electrical Nerve Stimulation (TENS) Affect EEG Gamma Band Activity? J Biomed Phys Eng. 2018 Sep 1;8(3):271-280. PMID: 30320031; PMCID: PMC6169118.
8. Equinox Group. (c. 1988). The Equinox System: Electro-stimulation. Module E3. Equinox, Liverpool.
9. Gao W, Zhang L, Han X, Wei L, Fang J, Zhang X, Zhang J, Wang H, Zhou Q, Wang C, Chen W, Ni X, Yang L, Du R, Wang G, Liu B, Li Y, Zhang S, Wang Q. Transcutaneous Electrical Acupoint Stimulation Decreases the Incidence of Postoperative Nausea and Vomiting After Laparoscopic Non-gastrointestinal Surgery: A Multi-Center

Randomized Controlled Trial. Front Med (Lausanne). 2022 Mar 14;9:766244. doi: 10.3389/fmed.2022.766244. PMID: 35360742; PMCID: PMC8964119.

10. Ge Y, Zheng J. The efficacy of transcutaneous electronic acupoint stimulation for improving postoperative recovery after gynecologic surgery: A systematic review and meta-analysis. Medicine (Baltimore). 2023 Sep 1;102(35):e34834. doi: 10.1097/MD.0000000000034834. PMID: 37657060; PMCID: PMC10476788.

11. Goldberger SM, Tursky B. Modulation of shock-elicited pain by acupuncture and suggestion. Pain. 1976 Dec 1;2(4):417-29.

12. Gong Y, Li J, Wu XK. [Transcutaneous electrical acupoint stimulation for the treatment of idiopathic oligoasthenospermia: A meta-analysis]. Zhonghua Nan Ke Xue. 2021 Oct 20;27(10):917-926. Chinese. PMID: 34914271.

13. Han JS. et al. The Neurochemical Basis of Pain Relief by Acupuncture: A Collection of Papers 1973-1987. Beijing Medical University, vol. 1, 597 pp, 1987

14. Han JS. et al. The Neurochemical Basis of Pain Relief by Acupuncture. Hubei Science and Technology Press, vol. 2, 783 pp, 1998

15. Han JS, Tang J, Huang BS, Liang XN, Zhang NH. Acupuncture tolerance in rats: anti-opiate substrates implicated. Chin Med J (Engl). 1979 Sep;92(9):625-7. PMID: 115645.

16. Han JS, Chen XH, Yuan Y, Yan SC. Transcutaneous electrical nerve stimulation for treatment of spinal spasticity. Chinese Medical Journal. 1994;107(01):6-11.

17. Hsu YC, Liang IT, Huang SY, Wang HS, Soong YK, Chang CL. Transcutaneous electrical acupoint stimulation (TEAS) treatment improves pregnancy rate and implantation rate in patients with implantation failure. Taiwan J Obstet Gynecol. 2017 Oct;56(5):672-676. doi: 10.1016/j.tjog.2017.08.017. PMID: 29037556.

18. Huang S, Peng W, Tian X, Liang H, Jia Z, Lo T, He M, Feng Y. Effects of transcutaneous electrical acupoint stimulation at different frequencies on perioperative anesthetic dosage, recovery, complications, and prognosis in video-assisted thoracic surgical lobectomy: a randomized, double-blinded, placebo-controlled trial. J Anesth. 2017 Feb;31(1):58-65. doi: 10.1007/s00540-015-2057-1. Epub 2015 Sep 8. PMID: 26350110.

19. Jesus FG, Duque AP, Sant C, Massolar A, Lopes GPF, Carvalho ACA, Mediano MFF, Rodrigues LF. Transcutaneous electrical nerve stimulation of PC5 and PC6 acupoints increases sympathovagal balance but not oxidative stress in healthy subjects: a randomized clinical trial. J Acupunct Meridian Stud. 2021 Oct 31;14(5):183-192. doi: 10.51507/j.jams.2021.14.5.183. PMID: 35770587.

20. Jiang Y, Hao Y, Zhang Y, Liu J, Wang X, Han J, Fang J, Zhang J, Cui C. Thirty minute transcutaneous electric acupoint stimulation modulates resting state brain activities:

a perfusion and BOLD fMRI study. Brain Res. 2012 May 31;1457:13-25. doi: 10.1016/j.brainres.2012.03.063. Epub 2012 Apr 3. PMID: 22541167. [fMRI]

21. Jiang Y, Wang H, Liu Z, Dong Y, Dong Y, Xiang X, Bai L, Tian J, Wu L, Han J, Cui C. Manipulation of and sustained effects on the human brain induced by different modalities of acupuncture: an fMRI study. PLoS One. 2013 Jun 28;8(6):e66815. doi: 10.1371/journal.pone.0066815. PMID: 23840533; PMCID: PMC3696086. [fMRI]

22. Jiang Y, Liu J, Liu J, Han J, Wang X, Cui C. Cerebral blood flow-based evidence for mechanisms of low- versus high-frequency transcutaneous electric acupoint stimulation analgesia: a perfusion fMRI study in humans. Neuroscience. 2014 May 30;268:180-93. doi: 10.1016/j.neuroscience.2014.03.019. Epub 2014 Mar 20. PMID: 24657460.

23. Jiang X, Shou XJ, Zhao Z, Chen Y, Meng FC, Le J, Song TJ, Xu XJ, Guo W, Ke X, Cai XE, Zhao W, Kou J, Huo R, Liu Y, Yuan HS, Xing Y, Han JS, Han SP, Li Y, Lai H, Zhang L, Jia MX, Liu J, Liu X, Kendrick KM, Zhang R. A brain structural connectivity biomarker for autism spectrum disorder diagnosis in early childhood. Psychoradiology. 2023 Apr 20;3:kkad005. doi: 10.1093/psyrad/kkad005. PMID: 38666122; PMCID: PMC11003421.

24. Jin ZR, Liu BH, Tang WH, Jiang H, Zhang R, Han JS, Xing GG. [Transcutaneous electrical acupoint stimulation for asthenozoospermia]. Zhonghua Nan Ke Xue. 2017 Jan;23(1):73-77. Chinese. PMID: 29658242.

25. Jin ZR, Fang D, Liu BH, Cai J, Tang WH, Jiang H, Xing GG. Roles of CatSper channels in the pathogenesis of asthenozoospermia and the therapeutic effects of acupuncture-like treatment on asthenozoospermia. Theranostics. 2021 Jan 1;11(6):2822-2844. doi: 10.7150/thno.51869. PMID: 33456575; PMCID: PMC7806476.

26. [a] Kaada B, Eielsen O. In search of mediators of skin vasodilation induced by transcutaneous nerve stimulation: I. Failure to block the response by antagonists of endogenous vasodilators. General Pharmacology: The Vascular System. 1983 Jan 1;14(6):623-33.

27. [b] Kaada B, Eielsen O. In search of mediators of skin vasodilation induced by transcutaneous nerve stimulation: II. Serotonin implicated. General Pharmacology: The Vascular System. 1983 Jan 1;14(6):635-41.

28. [c] Kaada B, Olsen E, Eielsen O. In search of mediators of skin vasodilation induced by transcutaneous nerve stimulation: III. Increase in plasma VIP in normal subjects and in Raynaud's disease. General Pharmacology. 1984 Jan 1;15(2):107-13.

29. [d] Kaada B, Helle KB. In search of mediators of skin vasodilation induced by transcutaneous nerve stimulation: IV. In vitro bioassay of the vasoinhibitory activity of sera from patients suffering from peripheral ischaemia. General Pharmacology. 1984 Jan 1;15(2):115-22.

30. Kaada B, Vik-Mo H, Rosland G, Woie L, Opstad PK. Transcutaneous nerve stimulation in patients with coronary arterial disease: haemodynamic and biochemical effects. European Heart Journal. 1990 May 1;11(5):447-53.

31. Kong SP, Tan QW, Liu Y, Jing XH, Zhu B, Huo YJ, Nie BB, Yang DH. Specific correlation between the hegu point (LI4) and the orofacial part: evidence from an fMRI study. Evid Based Complement Alternat Med. 2015;2015:585493. doi: 10.1155/2015/585493. Epub 2015 Sep 13. PMID: 26446439; PMCID: PMC4584065.

32. Li H, Wu C, Yan C, Zhao S, Yang S, Liu P, Liu X, Wang M, Wang X. Cardioprotective effect of transcutaneous electrical acupuncture point stimulation on perioperative elderly patients with coronary heart disease: a prospective, randomized, controlled clinical trial. Clin Interv Aging. 2019 Sep 6;14:1607-1614. doi: 10.2147/CIA.S210751. PMID: 31564843; PMCID: PMC6735632.

33. Li JJ, Zhao WS, Shao XM, Yang AM, Zhang FF, Fang JQ. [Effect of transcutaneous electrical acupoint stimulation on post-surgical gastrointestinal function, autonomic nerve activities and plasma brain-gut peptide levels in patients undergoing gastrointestinal surgery]. Zhen Ci Yan Jiu. 2016 Jun 25;41(3):240-6. Chinese. PMID: 29071913.

34. Li SZ. 2007 (Ed. Mayor D). Clinical Applications of Commonly Used Acupuncture Points. Potters Bar, Hertfordshire: Donica Publishing.

35. Liu X, Wang J, Wang B, Wang YH, Teng Q, Yan J, Wang S, Wan Y. Effect of transcutaneous acupoint electrical stimulation on propofol sedation: an electroencephalogram analysis of patients undergoing pituitary adenomas resection. BMC Complement Altern Med. 2016 Jan 27;16:33. doi: 10.1186/s12906-016-1008-1. PMID: 26817460; PMCID: PMC4729180.

36. Liu X, Gao Z, Jiang Y, Tuo X, He S, Xu F, Lu Z. Comparison of low-frequency or high-frequency electrical acupoint stimulation on hypotension after spinal anesthesia in parturients: a prospective randomized controlled clinical trial. J Integr Complement Med. 2024 Aug;30(8):770-775. doi: 10.1089/jicm.2023.0610. Epub 2024 Mar 28. PMID: 38546428.

37. Low HJ, Cheah OK, Ng BH, Siti Nidzwani MM, Wan Rahiza WM, Liu CY. Effect of transcutaneous electrical acupoint stimulation in heart rate variability in post-on-call trainees. Med J Malaysia. 2024 Nov;79(6):764-769. PMID: 39614796.

38. Lu C, Du JY, Fang JQ, Fang JF. [The curative effect observation of different frequency of TEAS combined with wristband pressing on neiguan (PC 6) for nausea and vomiting after laparoscopic cholecystectomy]. Zhongguo Zhen Jiu. 2019 Jan 12;39(1):9-15. Chinese. doi: 10.13703/j.0255-2930.2019.01.002. PMID: 30672249.

39. Ma LH, Zhang Z, Ma LX, Mu JD, Qian X, Zhang QY, Sun TY. Biofeedback physical regulation of hypertension based on acupoints: A clinical trial. Medicine (Baltimore).

2023 Jun 23;102(25):e33946. doi: 10.1097/MD.0000000000033946. PMID: 37352053; PMCID: PMC10289684.

40. Mayor DF. 2001. CNS resonances to peripheral stimulation: is frequency important? AACP/IAAPT Conference, Latimer, Bucks, UK, 24 March 2001.

41. Mayor DF (Ed.). 2007. Electroacupuncture: A practical manual and resource. Edinburgh: Churchill Livingstone (Elsevier).

42. Mayor D, Steffert T, Panday D, Noreikaite A, Zaleczna L. 2019. Does electrical stimulation to the hands (transcutaneous electroacupuncture stimulation, TEAS) have frequency-specific effects on heart rate variability (HRV)? Conference poster, ARRC Symposium, London. http://electroacupuncture.qeeg.co.uk/hrv2 [accessed 11 February 2025].

43. Mayor D, Panday D, Kandel HK, Steffert T, Banks D. CEPS: an open access MATLAB graphical user interface (GUI) for the analysis of complexity and entropy in physiological signals. Entropy (Basel). 2021 Mar 8;23(3):321. doi: 10.3390/e23030321. PMID: 33800469; PMCID: PMC7998823.

44. Mayor D, Steffert T, Datseris G, Firth A, Panday D, Kandel H, Banks D. Complexity and entropy in physiological signals (CEPS): resonance breathing rate assessed using measures of fractal dimension, heart rate asymmetry and permutation entropy. Entropy (Basel). 2023 Feb 6;25(2):301. doi: 10.3390/e25020301. PMID: 36832667; PMCID: PMC9955651.

45. Meade CS, Lukas SE, McDonald LJ, Fitzmaurice GM, Eldridge JA, Merrill N, Weiss RD. A randomized trial of transcutaneous electric acupoint stimulation as adjunctive treatment for opioid detoxification. J Subst Abuse Treat. 2010 Jan;38(1):12-21. doi: 10.1016/j.jsat.2009.05.010. Epub 2009 Jul 1. PMID: 19574017; PMCID: PMC2789908.

46. Melzack R, Wall PD. Pain mechanism: a new theory. Science 1965;150(3699):971–9.

47. Mi Z, Gao J, Chen X, Ge Y, Lu K. [Effects of transcutaneous electrical acupoint stimulation on quality of recovery during early period after laparoscopic cholecystectomy]. Zhongguo Zhen Jiu. 2018 Mar 12;38(3):256-60. Chinese. doi: 10.13703/j.0255-2930.2018.03.007. PMID: 29701042.

48. Moreira BR, Duque AP, Massolar CS, de Lima Pimentel R, Mediano MFF, Guimarães TCF, Rodrigues LF Jr. Transcutaneous electrical stimulation of PC5 and PC6 acupoints modulates autonomic balance in heart transplant patients: a pilot study. J Acupunct Meridian Stud. 2019 Jun;12(3):84-89. doi: 10.1016/j.jams.2019.04.001. Epub 2019 Apr 23. PMID: 31026520.

49. Penetar DM, Burgos-Robles A, Trksak GH, Maclean RR, Dunlap S, Lee DY, Lukas SE. Effects of transcutaneous electric acupoint stimulation on drug use and responses to cue-induced craving: a pilot study. Chin Med. 2012 Jun 10;7(1):14. doi: 10.1186/1749-8546-7-14. PMID: 22682006; PMCID: PMC3412709.

50. Pomeranz B, Niznick G. Codetron, a new electrotherapy device overcomes the habituation problems of conventional TENS devices. American Journal of Electromedicine. 1987; 2; 22-26.

51. Qi Y, Luo F, Zhang W, Wang Y, Chang J, Woodward DJ, Chen AC, Han J. Sliding-window technique for the analysis of cerebral evoked potentials. Beijing Da Xue Xue Bao Yi Xue Ban. 2003 Jun 18;35(3):231-5. PMID: 12914235. [EEG]

52. Qu F, Wang FF, Wu Y, Zhou J, Robinson N, Hardiman PJ, Pan JX, He YJ, Zhu YH, Wang HZ, Ye XQ, He KL, Cui L, Zhao HL, Ye YH. transcutaneous electrical acupoint stimulation improves the outcomes of in vitro fertilization: a prospective, randomized and controlled study. Explore (NY). 2017 Sep-Oct;13(5):306-312. doi: 10.1016/j.explore.2017.06.004. Epub 2017 Jun 30. PMID: 28915981.

53. Research Group of Acupuncture Anesthesia, Peking Medical College, Peking. Effect of acupuncture on pain threshold of human skin. Chinese Medical Journal. 1973; 86(3): 35. doi: 10.5555/cmj.0366-6999.86.03.p35.01.

54. Salansky N, Fedotchev A, Bondar A. Responses of the nervous system to low frequency stimulation and EEG rhythms: clinical implications. Neurosci Biobehav Rev. 1998 May;22(3):395-409. doi: 10.1016/s0149-7634(97)00029-8. PMID: 9579328.

55. Sherry JE, Oehrlein KM, Hegge KS, Morgan BJ. Effect of burst-mode transcutaneous electrical nerve stimulation on peripheral vascular resistance. Phys Ther. 2001 Jun;81(6):1183-91. PMID: 11380274.

56. Shou XJ, Xu XJ, Zeng XZ, Liu Y, Yuan HS, Xing Y, Jia MX, Wei QY, Han SP, Zhang R, Han JS. A volumetric and functional connectivity MRI study of brain arginine-vasopressin pathways in autistic children. Neurosci Bull. 2017 Apr;33(2):130-142. doi: 10.1007/s12264-017-0109-2. Epub 2017 Mar 3. PMID: 28258508; PMCID: PMC5360858.

57. Shuai Z, Li X, Tang X, Lian F, Sun Z. Transcutaneous electrical acupuncture point stimulation improves pregnancy outcomes in patients with recurrent implantation failure undergoing in vitro fertilisation and embryo transfer: a prospective, randomised trial. Acupunct Med. 2019 Feb;37(1):33-39. doi: 10.1136/acupmed-2017-011483. Epub 2019 Mar 13. PMID: 30864824.

58. Sjölund B, Terenius L, Eriksson M. Increased cerebrospinal fluid levels of endorphins after electro-acupuncture. Acta Physiologica Scandinavica. 1977 Jul;100(3):382-4.

59. So RC, Ng JK, Ng GY. Effect of transcutaneous electrical acupoint stimulation on fatigue recovery of the quadriceps. Eur J Appl Physiol. 2007 Aug;100(6):693-700. doi: 10.1007/s00421-007-0463-2. Epub 2007 Jun 2. PMID: 17546460.

60. Song Y, Xue X, Han H, Li C, Jian J, Yuan W, Chen X. Efficacy of transcutaneous electrical acupoint stimulation combined with diazepam for acute alcohol withdrawal syndrome: A double-blind randomized sham-controlled trial. J Int Med Res. 2020

Apr;48(4):300060520910052. doi: 10.1177/0300060520910052. PMID: 32340502; PMCID: PMC7218471.

61. Tu JF, Wang LQ, Liu JH, Qi YS, Tian ZX, Wang Y, Yang JW, Shi GX, Kang SB, Liu CZ. Home-based transcutaneous electrical acupoint stimulation for hypertension: a randomized controlled pilot trial. Hypertens Res. 2021 Oct;44(10):1300-1306. doi: 10.1038/s41440-021-00702-5. Epub 2021 Aug 6. PMID: 34363051; PMCID: PMC8342269.

62. Wang H, Xie Y, Zhang Q, Xu N, Zhong H, Dong H, Liu L, Jiang T, Wang Q, Xiong L. Transcutaneous electric acupoint stimulation reduces intra-operative remifentanil consumption and alleviates postoperative side-effects in patients undergoing sinusotomy: a prospective, randomized, placebo-controlled trial. Br J Anaesth. 2014 Jun;112(6):1075-82. doi: 10.1093/bja/aeu001. Epub 2014 Feb 26. PMID: 24576720.

63. Wang H, Yin N, Wang A, Xu G. Cerebral cortex functional networks of transdermal electrical stimulation at daling (PC7) acupoint. Clin EEG Neurosci. 2023 Mar;54(2):106-116. doi: 10.1177/15500594221123692. Epub 2022 Sep 15. PMID: 36113449.

64. Wang H, Xiang Y, Wang C, Wang Y, Chen S, Ding L, Liu Q, Wang X, Zhao K, Jia J, Chen Y. Effects of transcutaneous electrical acupoint stimulation on upper-limb impairment after stroke: A randomized, controlled, single-blind trial. Clin Rehabil. 2023 May;37(5):667-678. doi: 10.1177/02692155221138916. Epub 2022 Nov 15. PMID: 36380681; PMCID: PMC10041575.

65. Wang J, Wang J, Li X, Li D, Li XL, Han JS, Wan Y. Modulation of brain electroencephalography oscillations by electroacupuncture in a rat model of postincisional pain. Evid Based Complement Alternat Med. 2013;2013:160357. doi: 10.1155/2013/160357. Epub 2013 Apr 28. PMID: 23710210; PMCID: PMC3655616. [EEG]

66. Wang X, Yin L, Wang Y, Zhang H, Zhang S, Wu J, Fan S, Li Z, Li H, Wang J. Transcutaneous electrical acupoint stimulation for upper limb motor recovery after stroke: a systematic review and meta-analysis. Front Aging Neurosci. 2024 Nov 27;16:1438994. doi: 10.3389/fnagi.2024.1438994. PMID: 39665041; PMCID: PMC11631906.

67. Wei Y, Zheng Y. Transcutaneous electronic acupoint stimulation improves bone marrow suppression in lung cancer patients following chemotherapy: A systematic review and meta-analysis of randomized controlled trials. Medicine (Baltimore). 2023 Apr 21;102(16):e33571. doi: 10.1097/MD.0000000000033571. PMID: 37083807; PMCID: PMC10118342.

68. Wong RK, Jones GW, Sagar SM, Babjak AF, Whelan T. A Phase I-II study in the use of acupuncture-like transcutaneous nerve stimulation in the treatment of radiation-induced xerostomia in head-and-neck cancer patients treated with radical

radiotherapy. Int J Radiat Oncol Biol Phys. 2003 Oct 1;57(2):472-80. doi: 10.1016/s0360-3016(03)00572-8. PMID: 12957259.

69. Xiang XH, Chen YM, Zhang JM, Tian JH, Han JS, Cui CL. Low- and high-frequency transcutaneous electrical acupoint stimulation induces different effects on cerebral μ-opioid receptor availability in rhesus monkeys. J Neurosci Res. 2014 May;92(5):555-63. doi: 10.1002/jnr.23351. Epub 2014 Jan 31. PMID: 24482187. [PET]

70. Xu JJ, Ren M, Zhao JJ, Wu JJ, Zhang SC, Zhong YB, Xu ST, Cao ZY, Zhou ZQ, Li YL, Shan CL. Effectiveness of theta and gamma electroacupuncture for post-stroke patients on working memory and electrophysiology: study protocol for a double-center, randomized, patient- and assessor-blinded, sham-controlled, parallel, clinical trial. Trials. 2020 Nov 4;21(1):910. doi: 10.1186/s13063-020-04807-z. PMID: 33148333; PMCID: PMC7641837.

71. Yang H, Hu WH, Xu GX, Yin ZH, Yu SY, Liu JJ, Xiao ZY, Zheng XY, Yang J, Liang FR. Transcutaneous electrical acupoint stimulation for pregnancy outcomes in women undergoing *in vitro* fertilization-embryo transfer: A systematic review and meta-analysis. Front Public Health. 2022 Aug 11;10:892973. doi: 10.3389/fpubh.2022.892973. PMID: 36033802; PMCID: PMC9403762.

72. Yang LY, Lee BO, Lee KN, Chen CA. Effects of electrical stimulation of acupoints on xerostomia for patients who undergo hemodialysis. Healthcare (Basel). 2022 Mar 9;10(3):498. doi: 10.3390/healthcare10030498. PMID: 35326976; PMCID: PMC8954393.

73. Yang Y, Yang X, Dong Y, Chen N, Xiao X, Liu H, Li Z, Chen Y. Transcutaneous electrical acupoint stimulation alleviates adverse cardiac remodeling induced by overload training in rats. J Appl Physiol (1985). 2016 Jun 1;120(11):1269-76. doi: 10.1152/japplphysiol.00077.2016. Epub 2016 Mar 31. PMID: 27032900.

74. Yu X, Zhang F, Chen B. Effect of transcutaneous electrical acupuncture point stimulation at different frequencies in a rat model of neuropathic pain. Acupunct Med. 2017 Apr;35(2):142-147. doi: 10.1136/acupmed-2016-011063. Epub 2016 Oct 4. PMID: 27707699.

75. Yu Y, Sha SB, Zhang B, Guan Q, Liang M, Zhao LG, Zhang QY, Wen J, Sun W. Effects and mechanism of action of transcutaneous electrical acupuncture point stimulation in patients with abnormal semen parameters. Acupunct Med. 2019 Feb;37(1):25-32. doi: 10.1136/acupmed-2017-011365. Epub 2019 Apr 3. PMID: 30942613.

76. Zarei AA, Jensen W, Faghani Jadidi A, Lontis ER, Atashzar SF. Gamma-band enhancement of functional brain connectivity following transcutaneous electrical nerve stimulation. J Neural Eng. 2022 Mar 30;19(2). doi: 10.1088/1741-2552/ac59a1. PMID: 35234662.

77. Zhang W, Luo F, Qi Y, Wang Y, Chang J, Woodward DJ, Chen AC, Han J. Modulation of pain signal processing by electric acupoint stimulation: an electroencephalogram

study. Beijing Da Xue Xue Bao Yi Xue Ban. 2003 Jun 18;35(3):236-40. PMID: 12914236. [EEG]

78. Zhang WT, Jin Z, Cui GH, Zhang KL, Zhang L, Zeng YW, Luo F, Chen AC, Han JS. Relations between brain network activation and analgesic effect induced by low vs. high frequency electrical acupoint stimulation in different subjects: a functional magnetic resonance imaging study. Brain Res. 2003 Aug 29;982(2):168-78. doi: 10.1016/s0006-8993(03)02983-4. PMID: 12915252. [fMRI]

79. Zhang WT, Jin Z, Huang J, Zhang L, Zeng YW, Luo F, Chen AC, Han JS. Modulation of cold pain in human brain by electric acupoint stimulation: evidence from fMRI. Neuroreport. 2003 Aug 26;14(12):1591-6. doi: 10.1097/00001756-200308260-00010. PMID: 14502082. [fMRI]

80. Zhang WT, Jin Z, Luo F, Zhang L, Zeng YW, Han JS. Evidence from brain imaging with fMRI supporting functional specificity of acupoints in humans. Neurosci Lett. 2004 Jan 2;354(1):50-3. doi: 10.1016/j.neulet.2003.09.080. PMID: 14698480. [fMRI]

81. Zhang Y, Glielmi CB, Jiang Y, Wang J, Wang X, Fang J, Cui C, Han J, Hu X, Zhang J. Simultaneous CBF and BOLD mapping of high frequency acupuncture induced brain activity. Neurosci Lett. 2012 Nov 14;530(1):12-7. doi: 10.1016/j.neulet.2012.09.050. Epub 2012 Oct 4. PMID: 23041713.

82. Zhang Y, Jiang Y, Glielmi CB, Li L, Hu X, Wang X, Han J, Zhang J, Cui C, Fang J. Long-duration transcutaneous electric acupoint stimulation alters small-world brain functional networks. Magn Reson Imaging. 2013 Sep;31(7):1105-11. doi: 10.1016/j.mri.2013.01.006. Epub 2013 May 16. PMID: 23684242. [fMRI]

83. Zhao W, Wang C, Li Z, Chen L, Li J, Cui W, Ding S, Xi Q, Wang F, Jia F, Xiao S, Guo Y, Zhao Y. Efficacy and safety of transcutaneous electrical acupoint stimulation to treat muscle spasticity following brain injury: a double-blinded, multicenter, randomized controlled trial. PLoS One. 2015 Feb 2;10(2):e0116976. doi: 10.1371/journal.pone.0116976. PMID: 25643051; PMCID: PMC4314074.

84. Zhou D, Hu B, He S, Li X, Gong H, Li F, Wang Q. Transcutaneous Electrical Acupoint Stimulation Accelerates the Recovery of Gastrointestinal Function after Cesarean Section: A Randomized Controlled Trial. Evid Based Complement Alternat Med. 2018 Nov 13;2018:7341920. doi: 10.1155/2018/7341920. PMID: 30538764; PMCID: PMC6257894.

85. Zhu F, Zhao B, Wu J, Yin S, Ma T, Li Z, Zhu X, Wang T, Yang B, Che D. Effect of transcutaneous electrical acupoint stimulation on pregnancy outcomes in women with *in vitro* fertilization-embryo transfer: A systematic review and meta-analysis. Front Cell Dev Biol. 2022 Dec 12;10:1068894. doi: 10.3389/fcell.2022.1068894. PMID: 36578784; PMCID: PMC9791369.

86. Zhu SY, Ma J, Wang ZR, Chai Q, Yan LC, Song J, Shu JJ, Wang HM, Chen YD. EEG and ECG power spectrum analysis of sedative effects on propofol-anesthetized rats with

electroacupuncture. Evid Based Complement Alternat Med. 2022 May 27;2022:2440609. doi: 10.1155/2022/2440609. PMID: 35668776; PMCID: PMC9167085. [EEG]

87. Zuo YF, Wang JY, Chen JH, Qiao ZM, Han JS, Cui CL, Luo F. A comparison between spontaneous electroencephalographic activities induced by morphine and morphine-related environment in rats. Brain Res. 2007 Mar 9;1136(1):88-101. doi: 10.1016/j.brainres.2006.11.099. Epub 2006 Dec 20. PMID: 17234161. [EEG]
